# Guanidinate Yttrium Complexes Containing Bipyridyl and Bis(benzimidazolyl) Radicals

**DOI:** 10.1021/acs.inorgchem.4c00006

**Published:** 2024-04-03

**Authors:** Francis Delano, Saroshan Deshapriya, Selvan Demir

**Affiliations:** Department of Chemistry, Michigan State University (MSU), 578 South Shaw Lane, East Lansing, Michigan 48824, United States

## Abstract

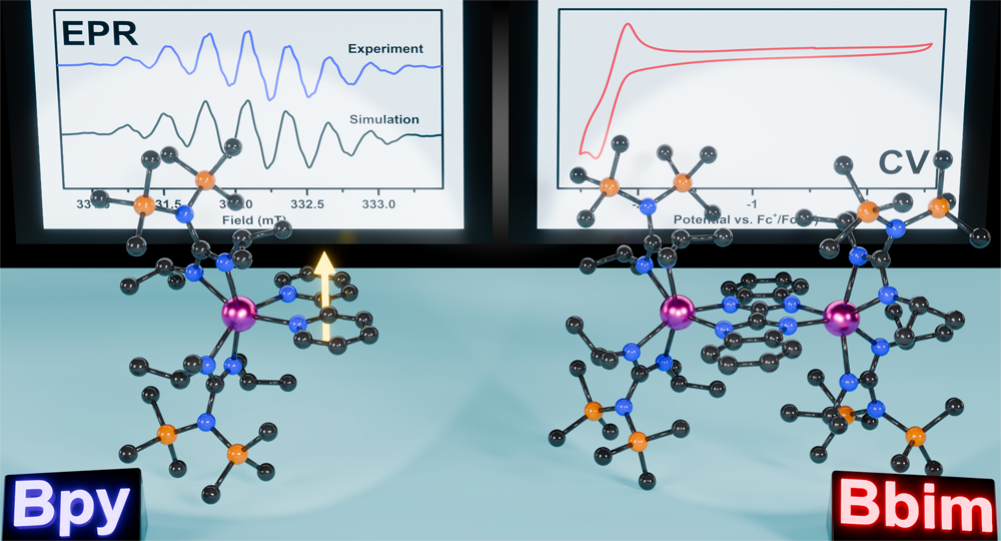

Ancillary ligand scaffolds that sufficiently stabilize a metal ion to allow its coordination to an open-shell ligand are scarce, yet their development is essential for next-generation spin-based materials with topical applications in quantum information science. To this end, a synthetic challenge must be met: devising molecules that enable the binding of a redox-active ligand through facile displacement and clean removal of a weakly coordinating anion. Here, we probe the accessibility of unprecedented radical-containing rare-earth guanidinate complexes by combining our recently discovered yttrium tetraphenylborate complex [{(Me_3_Si)_2_NC(N^i^Pr)_2_}_2_Y][(μ-η^6^-Ph)(BPh_3_)] with the redox-active ligands 2,2′-bipyridine (bpy) and 2,2′-bis(benzimidazole) (Bbim), respectively, under reductive conditions. Our endeavor resulted in the first evidence of guanidinate complexes that contain radicals, namely, a mononuclear bipyridyl radical complex, {(Me_3_Si)_2_NC(N^i^Pr)_2_}_2_Y(bpy^•^) (**1**), and a dinuclear bis(benzimidazolyl) radical-bridged complex, [K(crypt-222)][{(Me_3_Si)_2_NC(N^i^Pr)_2_}_2_Y]_2_(μ-Bbim^•^) (**2′**). The latter was achieved by an in situ reduction of [{(Me_3_Si)_2_NC(N^i^Pr)_2_}_2_Y]_2_(μ-Bbim) (**2**), which was isolated from a salt metathesis reaction. **1** and **2** were characterized by X-ray crystallography and IR and UV–vis spectroscopy. Variable-temperature electron paramagnetic resonance spectroscopy was applied to gain insight into the distribution of unpaired spin density on **1** and **2′**. Density functional theory calculations were conducted on **1** and **2′** to elucidate further their electronic structures. The redox activity of **1** and **2′** was also probed by electrochemical methods.

## Abstract 2

Unprecedented guanidinate rare-earth-metal complexes of mono- and dinuclear nature were isolated and characterized by crystallography, spectroscopy, and density functional theory computations. The first contains a 2,2′-bipyridine (bpy) radical anion, and the second bears a 2,2′-bis(benzimidazole) (Bbim) bridging dianion, which also serves to access only the second example of a Bbim radical anion through chemical reduction. The radical nature of the compounds was proven via continuous-wave electron paramagnetic resonance spectroscopy, and both compounds were additionally probed via cyclic voltammetry.

## Introduction

The development of flexible ligand platforms is highly desirable in coordination chemistry because discrete engineering of the primary coordination sphere of the metal ions, in particular rare-earth (RE) ions, constitutes an effective way to modulate both the physical and catalytic properties of the arising compounds.^1,2^ Among various ligands, N-based chelates are attractive molecular building blocks for RE complexes because they can be tailored for numerous applications.^3^ Amidinate, guanidinate, and substituted 1,3-diketiminate anions represent highly tunable ligands because both the steric and electronic contributions can be readily altered, impacting the stability, reactivity, and solubility of coordination compounds.^4^

An additional synthetic challenge to modulation of the metal’s coordination sphere is the pursuit of molecules containing open-shell ligands. The development of a synthetic approach to metal-radical compounds is highly intriguing because it allows access to attractive materials with stellar physical properties such as conductivity^5,6^ and magnetism^7^ that are exciting for various applications. In particular, adhering an open-shell ligand to a RE ion is extremely beneficial because the diffuse spin orbitals of the radical ligand can penetrate the deeply buried f-orbitals of the lanthanides.^7,8^ This engages the RE ion in more meaningful interactions with the ligand sphere compared to that with the implementation of a closed-shell ligand. In fact, interactions of this type can lead to strong magnetic exchange interactions and paired with strong magnetic anisotropy result in remarkable radical-bridged single-molecule magnets (SMMs).^7,9^ This effective yet rare approach to SMMs could be expanded to the development of complexes containing ancillary hexafluoro-2,4-pentanedionate,^10^ hydrotris(pyrazolyl)borate,^11^ bis(trimethylsilylamide),^12^ and cyclopentadienyl ligand scaffolds.^9,13,14^ SMMs are molecules that possess a thermal barrier to spin inversion ushering in slow magnetic relaxation, which in the absence of quantum tunneling, yields open magnetic hysteresis loops.^15−19^ This magnetic memory effect may be exploited in potential applications such as high-density information storage and spin-based electronics.^20−25^

The magnitude of the exchange coupling constant, *J*, signifying metal–radical interaction, is expected to be affected by the amount of spin density on the coordinating atoms of the radical where a larger spin density should result in stronger coupling.^7^ Systems with low spin density on the coordinating atoms of the radical ligand lead to weak magnetic exchange, particularly when used in conjunction with the contracted 4f-orbitals of the paramagnetic lanthanide ions. The presence of weak magnetic exchange coupling in multinuclear complexes typically engenders the type of SMM properties coined by fast magnetic relaxation and no magnetic memory, rendering them less suitable for applications such as high-density data storage.^26−29^ One strategy to enhance the magnitude of the magnetic exchange coupling is to implement highly electron-withdrawing ancillary ligands such as hexafluoroacetylacetonates, as shown in radical-containing complexes, which are proposed to augment the Lewis acidity of the metal ion.^7,30^ Excitingly, this suggests that the ancillary ligand scaffold is capable of influencing the interaction between the metal center and the spin density of the bridging ligand.

RE metal guanidinate complexes are notable for a wide array of applications, including catalysis,^4^ photocatalysis,^31^ and single-molecule magnetism.^32,33^ Intriguingly, the implementation of radical ligands into molecular systems bearing guanidinate anions has been hitherto unknown, which in itself is exciting to explore. More important to investigate is whether the electron-donating ability of these ancillary ligands impacts the strength of interaction between the radical and the metal center, which currently is unknown due to the lack of such examples. Apart from the potential influence on the spin-density distribution in complexes composed of open-shell bridging ligands, the electron-donating or -withdrawing ability of ancillary guanidinate ligands may change the relative energies of the molecular orbitals, thus giving rise to appealing spectroscopic features and changes in reduction potentials. Furthermore, such alterations to the electronic structure of a molecule may impact the reactivity and stability.

Herein, the synthesis and characterization of unprecedented radical complexes featuring guanidinate scaffolds is presented. Our investigations prompted the use of the redox-active ligands 2,2′-bipyridine (bpy) and 2,2′-bis(benzimidazole) (Bbim), which led to isolation of the mononuclear yttrium complex {(Me_3_Si)_2_NC(N^i^Pr)_2_}_2_Y(bpy^•^) (**1**), bearing a monoanionic bpy radical, and the dinuclear yttrium complex [{(Me_3_Si)_2_NC(N^i^Pr)_2_}_2_Y]_2_(μ-Bbim) (**2**), containing a Bbim dianion bridge. The structures of **1** and **2** were confirmed through single-crystal X-ray diffraction analysis. Density functional theory (DFT) calculations provided insight into the electronic structure. The chemical reduction of **2** afforded the corresponding Bbim radical-bridged complex [K(crypt-222)][{(Me_3_Si)_2_NC(N^i^Pr)_2_}_2_Y]_2_(μ-Bbim^•^) (**2′**). The spin distributions of **1** and **2′** were probed through electron paramagnetic resonance (EPR) spectroscopy and constitute seminal examples of the first guanidinate complexes featuring open-shell ligands for any metal ion.

## Experimental Section

### General Information

All manipulations described herein were performed under an inert N_2_ or Ar atmosphere with rigorous exclusion of oxygen and moisture by using Schlenk and glovebox techniques. House nitrogen was purified through a MBraun HP-500-MO-OX gas purifier prior to use. Toluene and *n*-pentane were dried by refluxing over potassium while diethylether (Et_2_O) was dried by refluxing over NaK alloy and distilled prior to use. *n*-Hexane and fluorobenzenewere dried by refluxing over calcium hydride and distilled prior to use. These solvents were tested for the presence of water and oxygen with a drop of sodium benzophenone radical solution in the glovebox. Anhydrous YCl_3_ and 2,2′-bipyridine (bpy) were purchased from Sigma-Aldrich and used without further purification. Diisopropylcarbodiimide was purchased from Alfa-Aesar and dried over 4 Å sieves prior to use. Lithium bis(trimethylsilyl)amide, LiN[Si(CH_3_)_3_]_2_, was purchased from Sigma-Aldrich, dissolved in toluene, filtered through a Celite plug, and recrystallized from toluene at −35 °C. 2.2.2-Cryptand (crypt-222) was purchased from Sigma-Aldrich and recrystallized from a hot *n*-hexane solution. Following a literature procedure, the lithium salt of the *N*,*N*′-diisopropyl-*N*′-bis(trimethylsilyl)guanidinate anion, Li[(Me_3_Si)_2_NC(N^i^Pr)_2_], was synthesized through the addition of LiN[Si(CH_3_)_3_]_2_ to an *n*-hexane solution of *N*,*N*′-diisopropylcarbodiimide.^34^ The potassium salt of the Bbim ligand, K_2_Bbim,^35^ was synthesized through deprotonation of H_2_Bbim.^36^ [HNEt_3_][BPh_4_],^37^ KC_8_,^38^ and [{(Me_3_Si)_2_NC(N^i^Pr)_2_}_2_Y][(μ-η^6^-Ph)(BPh_3_)]^33^ were prepared according to reported methods. A PerkinElmer 2400 Series II CHNS/O analyzer was used for the CHN elemental analyses. IR spectra were obtained through the use of a Cary 630 diamond ATR-IR spectrometer in a N_2_ atmosphere. UV–vis data were collected on an Agilent Cary 60 in a 1 cm cuvette equipped with a Schlenk adaptor. A PGSTAT204 potentiostat from Metrohm was used in an argon-filled glovebox for cyclic voltammetry (CV) measurements.

### Synthesis of {(Me_3_Si)_2_NC(N^i^Pr)_2_}_2_Y(bpy^•^) (**1**)

In a 20 mL scintillation vial, 15.4 mg (0.10 mmol) of bpy was added to a stirring 3 mL tetrahydrofuran (THF) solution containing 93.6 mg (0.10 mmol) of dissolved [{(Me_3_Si)_2_NC(N^i^Pr)_2_}_2_Y][(μ-η^6^-Ph)(BPh_3_)], resulting in an instant color change from colorless to yellow. After 5 min of stirring at 25 °C, 15.1 mg (0.11 mmol) of KC_8_ was added to the reaction at once, turning the solution color to a deep brown, accompanied by the formation of a gray solid. After 30 min of stirring, the reaction mixture was filtered through Celite and subsequently evaporated to dryness. The resulting brown solid was dissolved in 1 mL of hexane, filtered, and stored for crystallization at −35 °C. Brown single crystals of **1** suitable for single-crystal X-ray diffraction analysis were obtained in 24% crystalline yield (20.0 mg, 0.024 mmol). Anal. Calcd for C_36_H_72_N_8_Si_4_Y·0.5C_6_H_14_: C, 54.38; H, 9.24; N, 13.01. Found: C, 54.26; H, 9.01; N, 12.66. IR (ATR, cm^–1^): 2957m, 2925w, 2903w, 2866w, 1490m, 1435s, 1368m, 1317m, 1249s, 1202s, 1141w, 1051m, 938s, 819s, 758m, 719m, 678w.

### Synthesis of [{(Me_3_Si)_2_NC(N^i^Pr)_2_}_2_Y]_2_(μ-Bbim) (**2**)

230.8 mg (0.24 mmol) of [{(Me_3_Si)_2_NC(N^i^Pr)_2_}_2_Y][(μ-η^6^-Ph)(BPh_3_)] was suspended in 18 mL of Et_2_O in a 20 mL scintillation vial. To this, a 2 mL Et_2_O suspension containing 36.8 mg (0.12 mmol) of K_2_ Bbim was added dropwise. The cloudy reaction mixture was allowed to stir at room temperature for 2 h and subsequently filtered through a Celite plug. The light-yellow solution was evaporated to dryness, and the resulting foamy residue was redissolved in 3.5 mL of *n*-hexane. Colorless, insoluble solids were removed via filtration through Celite, affording a transparent light-yellow solution. All *n*-hexane was removed under vacuum, and the off-white solids (151.6 mg) were redissolved in 0.75 mL of pentane and stored at −35 °C for crystallization. Crystalline yield of **2**: 86.0 mg (0.06 mmol, 47%). ^1^H NMR (500 MHz, benzene-*d*_6_, 25 °C): δ 0.21 (s, 72 H, Si(CH_3_)_3_), 1.27 (br s, 48 H, CH(CH_3_)_2_), 3.84 (br s, 8 H, CH(CH_3_)_2_), 7.36 (dt, ^3^*J*_H–H_ = 3.40 and 6.81 Hz, 4 H, Bbim), 8.36 (dt, ^3^*J*_H–H_ = 3.46 and 7.02 Hz, 4 H, Bbim). ^13^C NMR (126 MHz, benzene-*d*_6_, 25 °C): δ 171.0 (CN_3_), 158.06 (Bbim), 144.09 (Bbim), 121.89 (Bbim), 118.57 (Bbim), 46.41 (CH(CH_3_)_2_), 27.62 (CH(CH_3_)_2_), 2.61 (Si(CH_3_)_3_). Anal. Calcd for C_66_H_136_N_16_Si_8_Y_2_·C_5_H_12_: C, 52.36; H, 9.16; N, 13.76. Found: C, 52.03; H, 9.31; N, 14.10. IR (ATR, cm^–1^): 2961m, 2924w, 2868w, 1610w, 1433s, 1365s, 1321s, 1250s, 1199s, 1139w, 1051s, 939s, 821s, 742m, 678w.

### Chemical Reduction of **2**

Crypt-222 (6.1 mg, 0.016 mmol) and KC_8_ (1.6 mg, 0.012 mmol) were added to a stirring 1 mL THF solution containing 25.0 mg (0.016 mmol) of **2** in a 20 mL scintillation vial. The reaction immediately progressed from colorless to deep blue-green, accompanied by the formation of black insoluble solids (presumably graphite). After 1 h of stirring, the reaction mixture was filtered to afford a dark blue-green solution. The filtrate was subsequently used to prepare two solutions for spectroscopic investigation: 2.0 mM and 74.8 μM for EPR and UV–vis spectroscopic analysis, respectively.

### X-ray Crystallography

Data were collected on an XtaLAB Synergy, Dualflex, and HyPix diffractometer using Cu Kα radiation. Brown block and colorless plate-shaped crystals of **1** and **2** with dimensions of 0.614 × 0.119 × 0.074 mm^3^ and 0.315 × 0.2 × 0.116 mm^3^, respectively, were suspended in *n*-paratone oil and mounted on a nylon loop. The temperature was controlled through the use of an Oxford Cryosystems low-temperature device, operating at *T* = 100.0(1) K.

In both cases, the data collection strategy, unit cell determination, and data reduction were performed by the *CrysAlisPro* software,^39^ which corrects for Lorentz polarization. Absorption effects were accounted for through the use of a numerical absorption correction based on Gaussian integration over a multifaceted crystal model using spherical harmonics implemented in the *SCALE3 ABSPACK*(40) scaling algorithm.

The structures were solved in the space groups *P*2_1_/*n* and *P*1̅ by using dual methods with *ShelXT*(41) and refined by least squares using version 2019/2 of *XL*(42) incorporated in *Olex2*.^43^ All non-H atoms were refined anisotropically. H-atom positions were calculated geometrically and refined by using the riding model.

### Computational Methods

All DFT calculations were performed with the *ORCA 5.0.3* package.^44,45^ The structure of **1** was optimized using the uTPSSh functional^46−48^ and the def2-TZVP basis set^49,50^ implemented through *ORCA*, employing Grimme’s D3 dispersion correction reformulated with Becke–Johnson damping (D3BJ).^51,52^ Crystal structure coordinates of **1** were used as a starting point for its optimization. The minimum structure was confirmed through analytical frequency calculations (Figure S16). Time-dependent DFT (TDDFT) calculations were carried out on the optimized structure of **1** for 150 excited states on the def2-TZVP level of theory using the unrestricted B3LYP functional^53,54^ with a CPCM implicit solvent model^55,56^ manually defined for Et_2_O. The calculated transitions were empirically shifted by 0.3 eV. Calculated coordinates of **2′** were generated by optimizing the crystal coordinates of **2** with a charge and a spin multiplicity of −1 and 2, respectively, at the def2-SVP level with the uTPSSh functional. The minimized structure was confirmed by a frequency calculation with no imaginary frequencies (Table S5). TDDFT states of **2′** were calculated with the uB3LYP functional at the def2-TZVP level using the CPCM THF solvent model. Molecular orbitals and spin-density information were generated using the Orca_plot module as cube files, and these were plotted using the *VMD* program.^57,58^

### EPR Spectroscopy

All EPR spectra were recorded on a Bruker EMX-plus spectrometer operating at X-band frequencies. The spectrometer is equipped with a Bruker ER4119HS probe and a modified Bruker liquid-nitrogen variable-temperature accessory. The data for **1** were collected under the following conditions: microwave frequency, 9.31 GHz; microwave power, 0.20 mW; field modulation amplitude, 0.01 mT. Samples were prepared in quartz EPR tubes using a 3 mM solution of **1** in thoroughly dried toluene. Spectra were collected at 298, 278, 258, 238, 218, 198, 178, and 158 K (Figure S14). Data for **2′** were collected with a 2 mM solution in THF at the same temperatures and similar conditions except for using a 0.006 mT modulation amplitude. All simulations were done using the *EasySpin 5.2.35* software package^59^ for *MATLAB*.

### CV Measurements

CV experiments were conducted under an inert atmosphere in an argon-filled glovebox. Data of **1** were measured using a PGSTAT204 potentiostat from Metrohm with a 2 mM sample solution in THF with [^n^Bu_4_N][BPh_4_] as the supporting electrolyte (100 mM) in conjunction with a glassy carbon working electrode, a Ag spring counter electrode, and a Ag wire pseudo reference electrode. The same setup was used for **2** and **2′**, with the measurements being carried out in 1 mM sample solutions in THF with the same supporting electrolyte concentration. All voltammograms were externally referenced to a ferrocene redox couple that was found to be 507 ± 57 mV, and all scans were conducted at 100 mV/s. To probe the influence of the solvent on the reversibility of the electrochemical behavior of **1**, a cyclic voltammogram was collected in fluorobenzene using [^n^Bu_4_N][PF_6_] as the supporting electrolyte, Figure S14.

## Results and Discussion

Ancillary ligand scaffolds that can stabilize a metal ion to allow its coordination to an open-shell ligand are synthetically challenging to achieve, in particular, for RE metal ions. This is attributed to the large size of an RE ion, rendering it more difficult to coordinatively saturate and stabilize when adjacent to a radical. Although such complexes represent challenging synthetic targets, molecules of this class have large ramifications in the field of quantum information science (QIS), in particular single-molecule magnetism.^20−25^ We introduce and explore the guanidinate scaffold class for this purpose. Thus, we set out to explore the feasibility of producing hitherto unknown guanidinate complexes that contain radical ligands, which will provide valuable structure–property relationships to advance QIS endeavors in the future. To this end, first, a generally applicable synthetic route to such coveted compounds must be devised. Our strategy to implement redox-active ligands into systems comprising ancillary guanidinate anions involves salt metathesis reactions where weakly coordinated [BPh_4_]^−^ ions are used. Recently, we reported the first guanidinate RE complexes featuring inner-sphere tetraphenylborate anions, [{(Me_3_Si)_2_NC(N^i^Pr)_2_}_2_RE][(μ-η^6^-Ph)(BPh_3_)] (RE = Y, Dy).^33^ Due to the steric hindrance imposed by the ancillary guanidinate ligands, the [BPh_4_]^−^ ion adopts an asymmetric coordination mode, leaving it weakly bound to the metal ion. Materials of this type are rare where the most prominent examples reside in cyclopentadienyl chemistry in the form of Cp^R^_2_REBPh_4_ (R = alkyl; Cp = cyclopentadienyl).^60,61^ Their utility has been demonstrated in reactions with various redox-active ligands, including 2,2′-azobis(pyridine),^62^ 2,2′-bipyrimidine,^8,63^ Bbim,^35^ phenazine,^60^ and tetrapyridylpyrazine,^64^ and ultimately enabled the isolation of radical-containing complexes.

To probe the generality of this synthetic approach to radical-containing compounds and expandability to systems with ancillary guanidinate ligands, we employed the redox-active bidentate ligand bpy and tetradentate ligand Bbim to generate guanidinate compounds comprising one and two metal centers, respectively. This approach has the added value of accessing compounds featuring radical-containing ligands of vastly different redox potentials.

**Figure 1:**
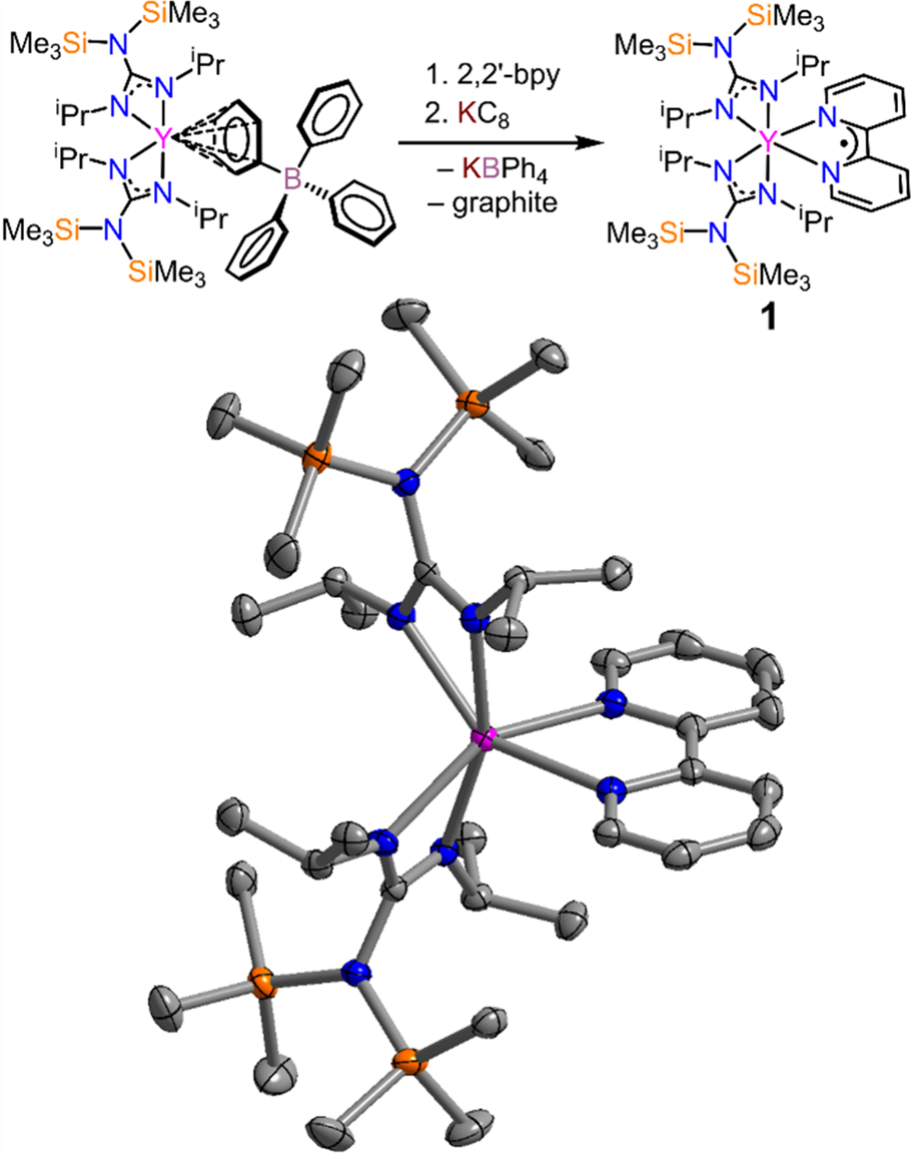
Synthetic scheme (upper) and structure of **1** (lower) in a crystal of **1**·C_6_H_14_, with thermal ellipsoids drawn at 50%. Pink, orange, blue, and gray ellipsoids represent the Y, Si, N, and C atoms, respectively. H atoms and cocrystallized hexane have been omitted for clarity.

**Figure 2:**
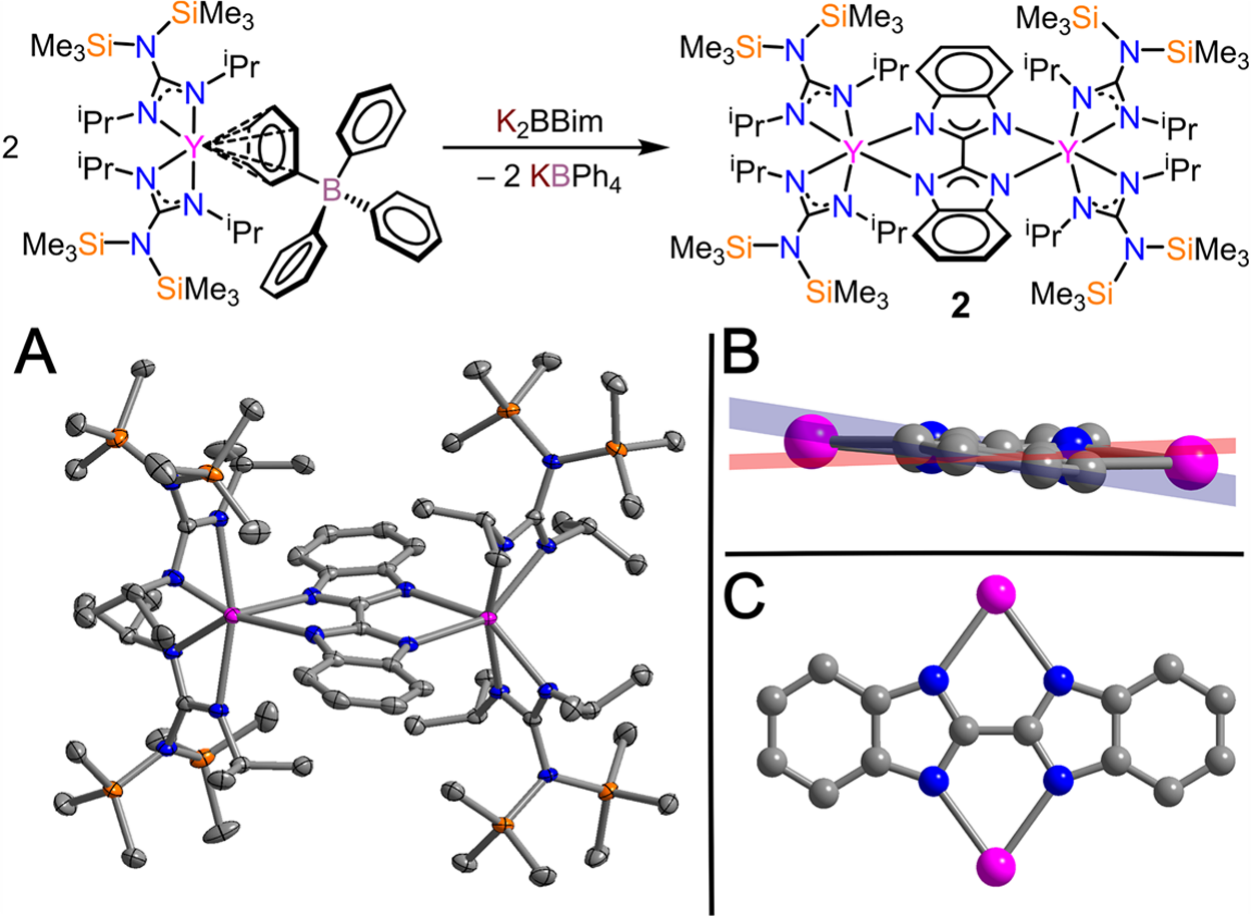
Top: Synthetic scheme for **2**. Bottom: (A) Structure of **2** in a crystal of **2**·2C_5_H_12_. Pink, orange, blue, and gray spheres represent Y, Si, N, and C atoms, respectively. H atoms and cocrystallized pentane have been omitted for clarity. (B) Magnification of the ligand core, highlighting the tilting of the Bbim ligand in the dianionic state upon complexation. (C) Depiction of the symmetric coordination of the Bbim ligand to the Y metal centers.

Monoanionic bpy ligands were first employed in the coordination chemistry of the RE metals in the 1960s, with the first examples being Ln^II^(bpy^•^)_2_(bpy)_2_ (Ln = Eu, Yb) complexes, bearing neutral and anionic bpy ligands, isolated from liquid ammonia solutions with the corresponding metal.^65^ Since then, ligands of this class have found extensive use in establishing the electronic structure of the RE metals,^66,67^ as well as establishing synthetic routes to achieve RE complexes through nontraditional synthetic pathways involving radical generations through H-atom-abstraction reactions.^68^ Thus far, only a handful of classes of L_2_RE(bpy^•^) complexes containing a bare bpy radical were discovered, with L ranging from trispyrazolylborates to bulky cyclopentadienyls to phospholyls (Figure S1), where none of these consist of yttrium ions.^66,69−74^ The use of yttrium(III) is advantageous because it possesses a nuclear spin of *I* = ^1^/_2_ and is diamagnetic. Thus, its combination with organic radicals enables profound insight into the electronic structure and spin-density distribution of a given complex. Notably, the only known yttrium bpy radical complexes are either homo- or heteroleptic, where all are composed of substituted bpy (Figure S1).^67,75^ The low-lying π* molecular orbital of bpy can be readily populated by one or two electrons, thus rendering it a suitable foundation to probe the accessibility of metal-radical complexes where the RE ion is stabilized by the guanidinate ligand platform. To test the generality of this approach, we extend this methodology to systems exceeding one nucleus by using Bbim, which is able to form bridged compounds in the dianionic state. The number of studies with Bbim in coordination chemistry is far less than that with bpy, where the first dates back to 1978.^76^ Very recently, the Demir group produced the first example of a trianionic paramagnetic state of Bbim^3–•^, encased between two metallocene cations of the type {Cp*_2_RE}^+^. The isolation of the previously elusive radical state was accomplished by taking advantage of the lowering of the lowest unoccupied molecular orbital (LUMO) of Bbim upon coordination to the metallocene cation.^13,35^ In addition to the influence of the metallocene unit on the molecular orbitals of the bridging ligand, DFT calculations uncovered the importance of the fused benzyl rings in the backbone of Bbim for access of its open-shell state because their absence precluded a stable paramagnetic species, which was the outcome of studies on the 2,2′-bis(imidazole)-bridged congeners.^77^ The lack of other Bbim^3–•^ radical anions highlights its challenging isolation and emphasizes the role of the ligated cationic unit. Thus, we were inspired to probe its accessibility in the realm of a guanidinium RE scaffold. The synthetic sequence used to obtain **1** and **2** utilizes both respective guanidinate yttrium tetraphenylborate complexes and differs in that one represents a salt elimination in the wake of a reduction path and the other constitutes a salt metathesis route (Figures 1 and 2). Both will be discussed below independently for clarity purposes.

The guanidinate yttrium bpy radical complex **1** was synthesized from the reaction of bpy with the guanidinate yttrium tetraphenylborate complex [{(Me_3_Si)_2_NC(N^i^Pr)_2_}_2_Y][(μ-η^6^-Ph)(BPh_3_)], followed by a one-electron chemical reduction employing the strong reducing agent potassium graphite (Figure 1). **1** was crystallized from a concentrated *n*-hexane solution at −35 °C in 24% crystalline yield. **1** crystallizes in the monoclinic space group *P*2_1_/*n* with four molecules in the unit cell (Figure S3) and constitutes the first crystallographically characterized guanidinate complex featuring a radical ligand, for any metal ion.

The six-coordinate Y^III^ center is ligated by two guanidinate anions and a bpy radical, resulting in a distorted octahedral geometry. The guanidinate ligands coordinate asymmetrically to the metal center, with bond distances ranging from 2.334(2) to 2.369(2) Å. The asymmetric coordination of ancillary guanidinate ligands has been observed in both mono- and dinuclear guanidinate RE complexes and is attributed to the resonance structures of the guanidinate ligand.^33^ The RE–N_guan_ distances are slightly shorter than the RE–N_bpy_ distances of 2.393(2) and 2.394(2) Å (where N_guan_ denotes the N atoms of the guanidinate ligand, and N_bpy_ denotes the N atoms of the bpy ligand). This decreased distance is ascribed to the larger delocalization of the negative charge in the bpy ligand. The C–RE–C angle, where C represents the central carbon on each guanidinate ligand, is 131.0(1)°, substantially larger than the analogous angle of the parental [BPh_4_]^−^ complex with 120.4(5)°,^33^ attributed to alleviation of the steric bulk resulting from the displacement of the [BPh_4_]^−^ moiety.

The crystallographic indication of a reduced bpy arising from the chemical addition of an electron using an alkali metal is best observed in a shortened interatomic distance of the central C–C bond between the two pyridyl rings.^78^ The central C–C distance in **1** is with 1.427(3) Å approximately 0.06 Å shorter compared to 1.49 Å in neutral bpy.^78,79^ The remaining interatomic distances within the reduced bpy ligand deviate slightly relative to free bpy (Figure S2B).^78^ In summary, all metrical parameters within the bpy unit hint at an overall monoanionic, reduced ligand.

The treatment of [{(Me_3_Si)_2_NC(N^i^Pr)_2_}_2_Y][(μ-η^6^-Ph)(BPh_3_)] with K_2_Bbim in Et_2_O afforded the first homoleptic bis(benzimidazolyl)-bridged yttrium guanidinate complex **2** (Figure 2). This also represents the second neutral yttrium complex that exhibits a Bbim ligand.^35^ Notably, the only other homoleptic neutral RE complexes constitute bis(benzimidazolyl)-bridged metallocene complexes, which were also isolated with Gd, Tb, and Dy.^13,35^**2** was crystallized from a concentrated *n*-pentane solution at −35 °C in 47% crystalline yield.

**2** crystallizes in the triclinic space group *P*1̅ with two molecules in the unit cell (Figure S7). The dinuclear complex features two six-coordinate Y^III^ centers, bridged by a dianionic Bbim^2–^ ligand. Similar to **1**, the guanidinate ions in **2** coordinate asymmetrically to the metal center, with interatomic distances spanning from 2.330(3) to 2.365(2) Å.

Interestingly, the Y–N_Bbim_ distance (N_bbim_ denotes the N atoms of the Bbim ligand) is 2.436(2) Å, considerably longer than 2.366(1) and 2.412(3) Å observed for the respective metallocene complexes (Cp*_2_Y)_2_(μ-Bbim), which may be the result of a larger steric bulk imposed by the guanidinate ions. This also causes a slightly larger intermetallic distance of 6.231(1) Å in **2** relative to 6.214(1) Å in (Cp*_2_Y)_2_(μ-Bbim). The two Y^III^ ions in **2** are inequivalent and feature different C–RE–C angles of 132.37(8) and 132.25(8)°, both of which are slightly larger than that observed for **1**.

Similar to **1**, the central C_imd_–C_imd_ (where C_imd_ is the bridging carbon of the imidazole ring) distance can act as a reporter for the charge of the bridging Bbim ligand, where successive reduction causes an elongation of the bond due to population of the vacant π* orbital.^8,35^ The central C_imd_–C_imd_ distance of **2** is 1.457(3) Å, consistent with the presence of a dianionic bridging ligand.^35^ Unexpectedly, a substantive torsion of the Bbim^2–^ ligand is observed in **2** (Figure 2B). The angle between the planes defined by the central C and N atoms of each benzimidazole unit is 10.7(1)°. This unprecedented torsion of the Bbim^2–^ ligand is attributed to the proximity of the isopropyl groups of the ancillary guanidinate anion (Figure S6). The distances between the centroids of the imidazole unit and the C atoms of the isopropyl substituent are 3.796(4) and 3.725(4) Å, respectively.

To probe the chemical accessibility of the radical oxidation state, the bis(benzimidazolyl)-bridged complex **2** was treated with 1 equiv of potassium graphite in the presence of crypt-222, resulting in a deep-blue-green solution. The one-electron reduction of an organometallic RE complex containing the Bbim^2–^ moiety yielded the corresponding trianionic radical state.^35^ The color of the reduction product is consistent with what is described for the generation of (Cp*_2_Y)_2_(μ-Bbim)^•–^ and indicative of the targeted radical-bridged compound, henceforth referred to as **2′**. It is expected that the high solubility arising from the trimethlylsilyl-substituted guanidinate ligands, coupled with the high reactivity of the targeted radical-bridged species, hitherto precluded crystal growth for X-ray diffraction studies. Despite the synthetic challenges, solutions of the *in situ* reduction product were analyzed to gain insight into the new radical species.

### Electrochemical Studies

**Figure 3:**
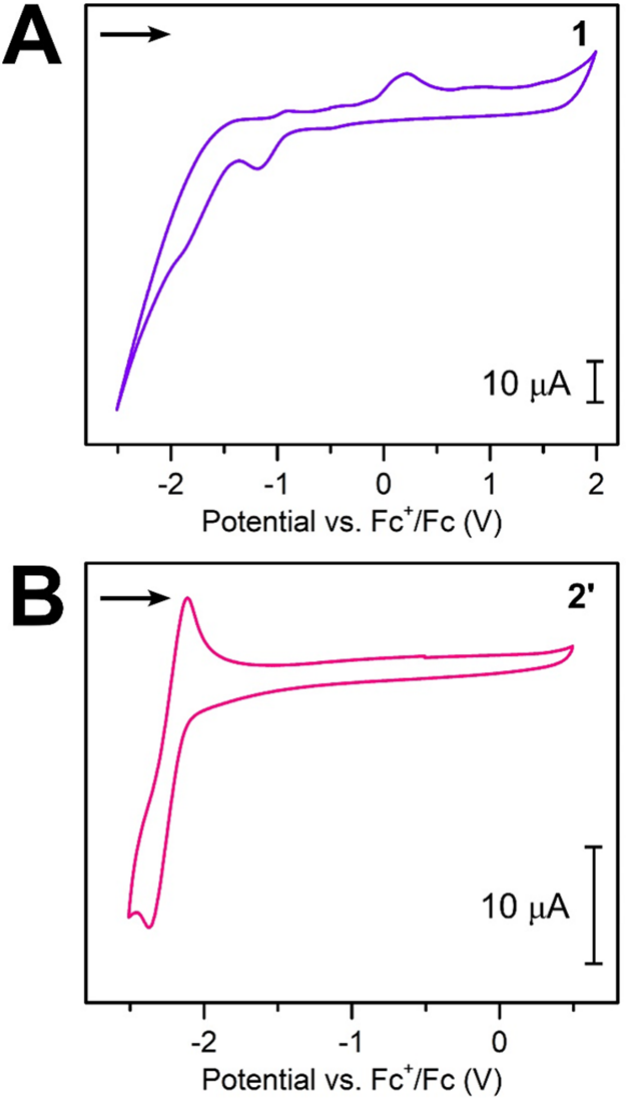
Cyclic voltammograms of (A) **1** and (B) **2′** vs Fc. Measurements were taken in 100 mM [^*n*^Bu_4_N][BPh_4_] in THF with analyte concentrations of 2 and 1 mM for **1** and **2′**, respectively.

The electrochemical behaviors of both **1** and **2′** were probed via CV using 2 and 1 mM THF solutions containing a 100 mM [^n^Bu_4_N][BPh_4_] supporting electrolyte. The cyclic voltammogram of **1** shows two irreversible redox events centered at −0.14(5) and −1.05(5) V vs ferrocenium/ferrocene (Fc^+^/Fc; Figure 3). These features are tentatively assigned to the bpy^0/–•^ and bpy^–•/2–^ redox couples, respectively. Notably, the uncoordinated neutral bpy ligand typically exhibits two chemically and electrochemically reversible features centered at −1.69 and −2.29 V vs Fc^+^/Fc,^80^ highlighting the influence that the coordinated metal unit has on the redox activity of the ligand. To probe the effects of the solvent on the electrochemical behavior of **1**, CV was performed in fluorobenzene using [^n^Bu_4_N][PF_6_] as the supporting electrolyte (Figure S14). The previously observed irreversible redox events corresponding to bpy^0/–•^ and bpy^–•/2–^ were monitored as quasi-reversible features at −0.03(3) and −1.81(3) V, respectively, indicating that the identity of the solvent has a small effect on the reversibility of the electrochemical redox events of this compound.

Unlike the redox behavior of free bpy, the redox activity of compounds containing Bbim derivatives is not as well investigated. Electrochemical measurements on the free H_2_Bbim compound or complexes containing the dianionic Bbim^2–^ unit lack any reversible or quasi-reversible features, which were previously attributed to the general redox inactivity of complexes containing H_2_Bbim or Bbim^2–^.^76,81,82^ In agreement with our previous example supported by two metallocenium cations, we did not observe any relevant redox events for the Bbim^2–^ unit of **2** (Figure S15); therefore, we probed the electrochemical behavior of **2′**. The CV of **2′** shows a quasi-reversible redox event at −2.24(5) V vs Fc^+^/Fc. The presence of a single redox feature agrees well with that observed for [(Cp*_2_Y)_2_(μ-Bbim^•^)]^−^ and has previously been assigned to the Bbim^3–•^/^4–^ redox couple.^35^ Notably, the observed redox feature of **2′** is cathodically shifted by 0.94 V in comparison to that of the analogous metallocene complex, – 2.24(5) and −1.30(7) vs Fc^+^/Fc for **2′** and [(Cp*_2_Y)_2_(μ-Bbim^•^)]^−^, respectively.^35^ Although redox-active ligands other than Bbim have shown shifts to less reducing potentials upon complexation, such a large alteration to the observed electrochemical behavior of the same ligand is remarkable because it suggests that the ancillary ligand platform plays a substantial role in the redox activity of the noninnocent ligands of RE metal systems. Although the RE ions are isolable in unusual oxidation states such as 2+ or 4+, they are extremely challenging to access due to the high reactivity of these oxidation states.^83−85^ The potential of the Y^III^/Y^II^ couple in (C_5_H_4_SiMe_3_)_3_Y is −3.04 V vs Fc^+^/Fc, significantly more reducing than that monitored for **2′**.^86^ Thus, the observed redox event is attributed to the bridging ligand rather than the metal. The discrepancy in the redox potentials may arise from a higher donating ability of guanidinate ligands relative to cyclopentadienyl anions, which could accumulate more electron density toward the bridge, rendering it more difficult to reduce.

### Spectroscopic Characterization

**Figure 4:**
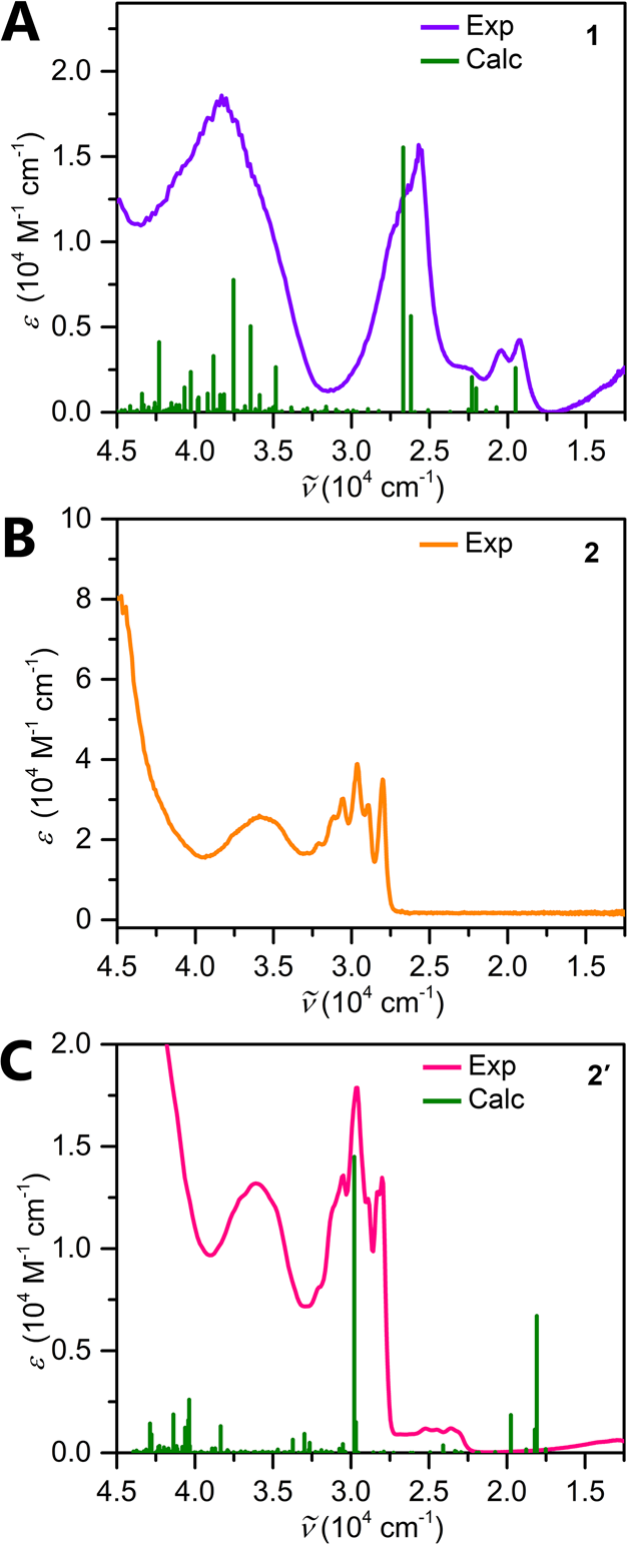
(A) UV–vis absorption spectrum of **1** in a Et_2_O solution. The violet line represents the experimental data for **1**, whereas the green line constitutes the calculated TDDFT transitions. (B) UV–vis spectrum of **2** taken in a Et_2_O solution. (C) UV–vis spectrum of **2′**, the product of the reduction of **2**, taken in a THF solution at approximately 75 μM.

The UV–vis absorption spectrum of **1** was collected from 250 to 1000 nm (Figure 4A). The absorption spectrum exhibits multiple transitions in the UV and visible regions, in accordance with numerous π–π* transitions arising from the different ligands ligated to the metal center. Complexes featuring bpy radical anions have intense transitions in the 700–1000 nm range, diagnostic of the open-shell state of the ligand, which is observed as a broad low-energy absorption band in the case of **1**.^73^ The identities of these transitions were confirmed through TDDFT computations of the optimized structure of **1**. The calculated transitions are plotted alongside the experimental absorption spectra in Figure 4A, and detailed information about the intense transitions is tabulated in Table S2. The most intense band is located at 374 nm and is primarily comprised of a ligand-to-ligand charge transfer (LLCT) originating from the spin-carrying bpy ligand to a molecular orbital composed of the bpy and ancillary guanidinate ligands with some contributions from the Y center. A ligand-to-metal charge transfer (LMCT) has a 29% contribution to this transition and mainly arises from the bpy ligand to the central Y^III^ ion. Remarkably, there are multiple transitions originating to and from the singly occupied molecular orbital (SOMO) throughout the electronic absorption spectra. The intense transition at 287 nm is primarily comprised of a LMCT arising from the ancillary guanidinate scaffold to the central Y ion.

The spectra of **2** and **2′** exhibit intense transitions in the UV region, which are attributed to a LMCT between the ancillary guanidinate ligands and central Y^III^ ion (Figure 4B,C). In addition to high-energy transitions, the UV–vis spectrum of **2′** exhibits absorbance bands in the visible (396, 408, and 423 nm) and near-IR regions of the spectrum, in accordance with the colored appearance of **2′**. To ascertain the identities of these transitions, TDDFT calculations were carried out on the optimized structure of **2′**. The most intense calculated transition corresponds to an LLCT from HOMO to LUMO originating from a primarily Bbim-based molecular orbital. The second most intense band also corresponds to an LLCT centered around Bbim. In addition, LMCTs are observed as less intense transitions arising from the ancillary guanidinates and Bbim as well. Detailed information about these transitions and the frontier orbitals involved is depicted in Table S3.

**Figure 5:**
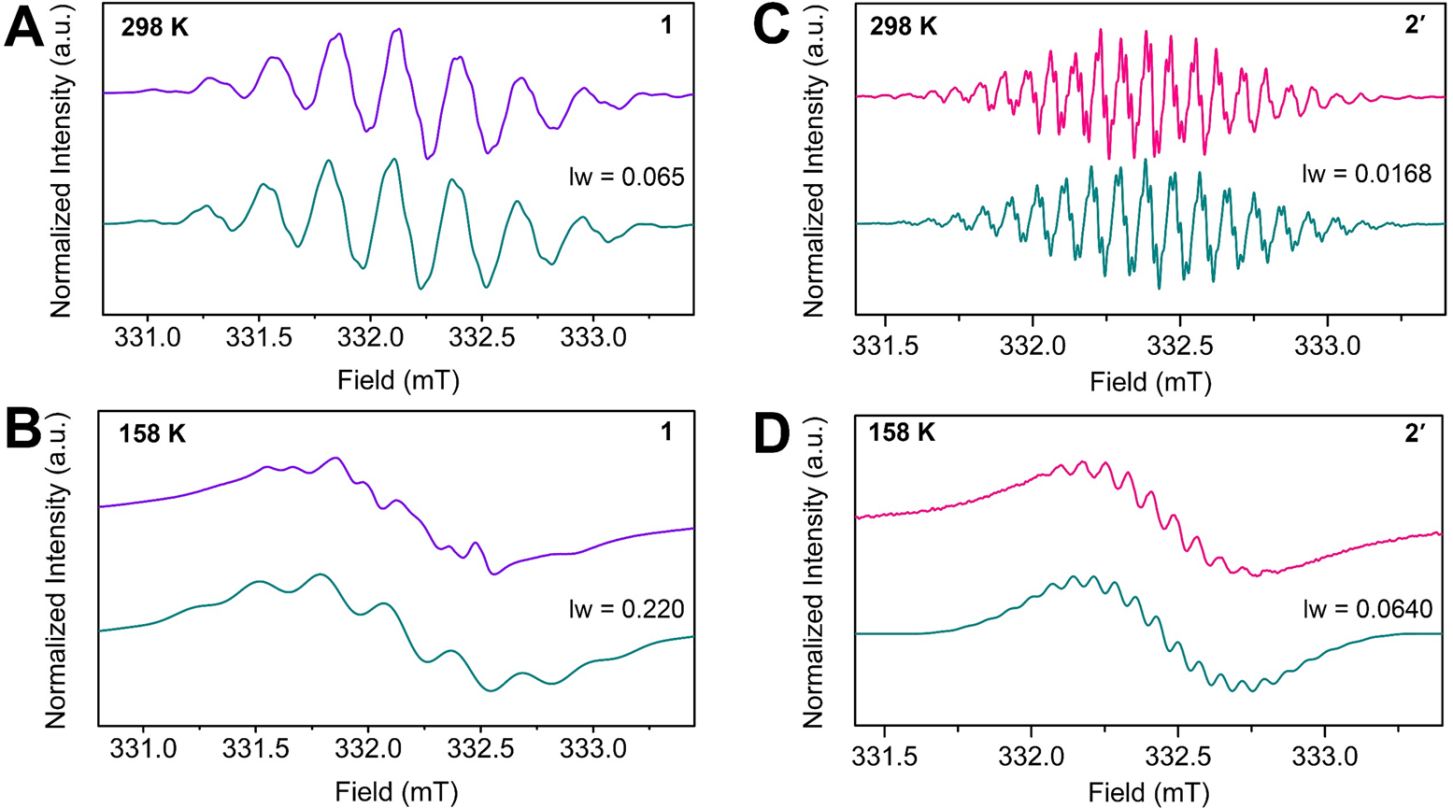
X-band cw-EPR spectra of **1** in toluene at (A) 298 and (B) 158 K and **2′** in THF at (C) 298 and (D) 158 K, respectively. The violet and pink lines represent the experimental data for **1** and **2′**, whereas the teal lines constitute the simulated EPR spectra with the line widths used for the simulations. Simulation data for **1**: spin system of four ^14^N, one ^89^Y, four ^1^H, and four ^1^H nuclei, *A*(^14^N) = 8.31 MHz, *A*(^89^Y) = 1.36 MHz, *A*_1_(^1^H) = 6.90 MHz, *A*_2_(^1^H) = 1.90 MHz, *g* = 2.0018, and line width (lw) = 0.065 at 298 K and 0.22 at 158 K. Simulation data for **2′**: spin system of four ^14^N, two ^89^Y, four ^1^H, and four ^1^H nuclei, *A*(^14^N) = 5.15 MHz, *A*(^89^Y) = 0.46 MHz, *A*_1_(^1^H) = 2.39 MHz, *A*_2_(^1^H) = 0.54 MHz, *g* = 2.0032, and lw = 0.0168 at 298 K and 0.064 at 158 K.

Excitingly, both the positions and assignments of these transitions are consistent with those observed for [(Cp*_2_Y)_2_(μ-Bbim^•^)]^−^ and support the assignment of a Bbim-centered radical. To probe the electronic structures further, variable-temperature X-band continuous-wave EPR (cw-EPR) spectra were experimentally collected and simulated for **1** and **2′** from 298 to 158 K in 20 K temperature increments (Figure S13). When spectra are collected at several different temperatures, the experimental hyperfine couplings can be more accurately determined because they can be simulated for multiple temperature regimes. The intensity-normalized cw-EPR spectrum of **1** consists of seven main lines, which were simulated with hyperfine couplings primarily stemming from N and H nuclei of the bpy ligand (Figure 5A).

**Figure 6:**
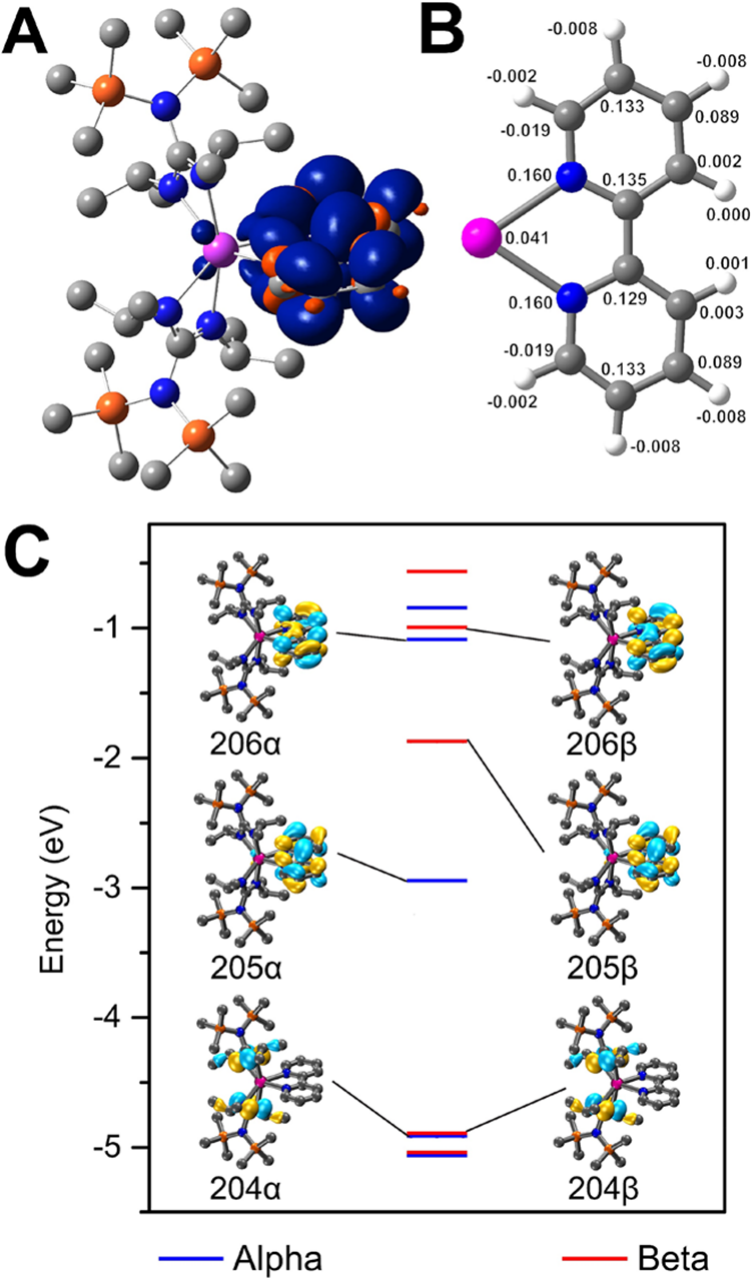
(A) DFT-calculated spin-density map of **1**. (B) Average calculated Mulliken spin densities for **1**. (C) Frontier orbitals of the optimized structure of **1**. The molecular orbital numbers 204, 205, and 206 correspond to HOMO, SOMO, and LUMO, respectively. Pink, gray, blue, and orange spheres represent Y, C, N, and Si atoms. H atoms have been omitted for the sake of clarity. Energy levels are shown to scale.

This description agrees well with the DFT calculations because the spin density is predominantly located on the bpy ligand with small displacement onto the Y center (Figure 6A). The *g* value of 2.0018 was determined based on the simulations for this and is within the vicinity of that for a free electron (*g* = 2.0023),^87^ further proving a distribution of the spin across the organic bpy radical moiety. Upon lowering of the temperature, features originating from the hyperfine coupling of the yttrium nucleus become more prevalent, as shown in Figure S13. At 158 K, which is well below the melting point of toluene (*M*_p_ = 178.2 K), the hyperfine couplings are not well resolved due to the considerable change in both *g* and *A* anisotropy. This is attributed to decreased molecular motions as the molecules are immobilized in the frozen solution. The cw-EPR spectrum of **2′** at 298 K consists of 19 main lines and was simulated predominantly with hyperfine coupling constants that correspond to N and H atoms of the Bbim ligand. In addition, smaller hyperfine coupling constants from Y centers were used. Variable-temperature EPR spectra exhibit a gradual loss of resolution with lowering temperature, which is again attributed to diminished molecular motion (Figure S13). The simulation of this spectrum was carried out with a *g* value of 2.0032, which is very close to that of the free electron, suggesting that the strongest interactions of the unpaired electron were within the Bbim ligand. The EPR spectrum is comparable to that of the only other yttrium compound containing a Bbim-centered radical, [K(crypt-222)][(Cp*_2_Y)(μ-Bbim^•^)].^35^ This included the employed hyperfine coupling constants, which in both compounds resemble one another (Figures 5C,D and S13). This picture is expected to differ for congeners incorporating paramagnetic metals due to the introduced magnetic anisotropy imposed by the distinct crystal fields.

### Computational Studies

The d^0^ configuration of the Y^III^ ion simplifies the investigation of the electronic structures of **1** and **2′** through DFT. **1** was optimized using the uTPSSh meta-GGA functional,^46−48^ and def2-TZVP basis set,^49,50^ employing Grimme’s D3 dispersion correction with Becke–Johnson damping.^51,52^ The relevant molecular orbitals of both the α- and β-spin manifolds of **1** are shown in Figure 6C. The highest occupied molecular orbital (HOMO) resides on the guanidinate ligands. In contrast, the SOMO is primarily composed of the bpy ligand and contains a bonding interaction between the bridging C–C bond of the two pyridyl rings. This supports the experimentally confirmed reduction of this interatomic distance relative to that of the free ligand. The LUMO also resides on the bpy ligand and validates the assignment of the redox features present in the cyclic voltammogram. To study the electronic structure of **2′**, crystal coordinates of **2** were optimized with a charge and a spin multiplicity of −1 and 2, respectively. The optimization was performed using the uTPSSh functional at the def2-SVP level, employing D3BJ dispersion correction.^51^ In contrast to **1**, both HOMO and SOMO of **2′** are primarily located on the bridging Bbim moiety and display no significant distribution into the ancillary guanidinate ligand scaffold. Notably, the LUMO arises from the guanidinate ligand orbitals, as well as the metal centers and the bridging Bbim ligand. These frontier orbitals are depicted in Figure S17 with their relative energies.

**Figure 7:**
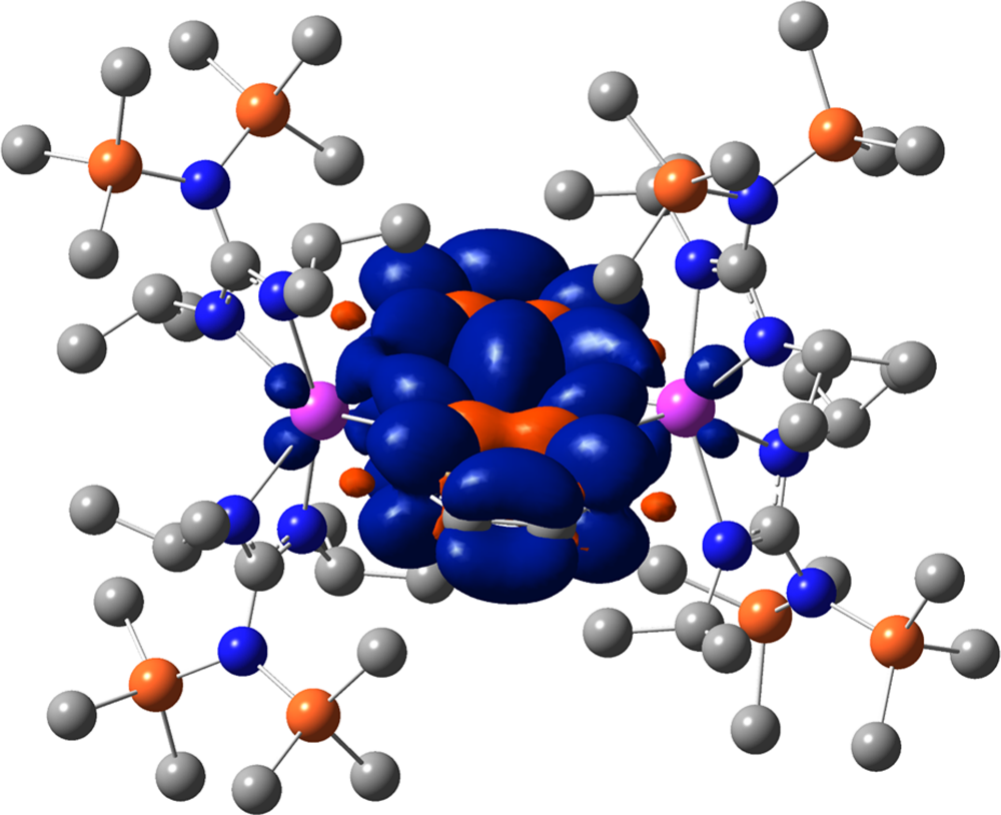
Calculated spin-density map of **2′**. Pink, blue, orange, and gray spheres represent Y, N, Si, and C atoms, respectively. H atoms have been omitted for clarity.

The calculated spin densities of both **1** and **2′** are displayed in Figures 6A and 7, respectively. In both cases, the unpaired electron spin resides predominantly on the aromatic ligand, with a small contribution from the central Y^III^ ion. This is congruent with the distribution of spin density attained through simulation of the EPR spectrum. Notably, relatively smaller metal-centered hyperfine couplings are required to appropriately simulate the experimentally acquired spectrum for **1**, hinting at a minor contribution of the metal ion to the overall spin-density distribution.

The relative energies of the computationally predicted molecular orbitals can provide insight into the experimentally determined electrochemical behaviors of the two radical compounds **1** and **2′**. The HOMO–SOMO gap of **1** has a 1.97 eV energy difference, whereas **2′** exhibits a significantly larger gap of 2.79 eV. The larger energy difference in **2′** is consistent with the absence of redox events corresponding to the Bbim^2–^/^3–•^ redox couple on the cyclic voltammogram of **2**.

## Conclusions

For the first time, the viability of the guanidinate yttrium tetraphenylborate complex in the synthesis of radical-containing compounds was demonstrated. First, the mononuclear complex **1** was isolated, which features an open-shell bpy ligand. The radical nature of the bpy ligand was proven by X-ray crystallography, EPR spectroscopy, and DFT computations. **1** represents the first crystallographically characterized guanidinate complex that contains a radical ligand for any metal ion. Second, through a salt metathesis route, the isolation of a rare bis(benzimidazolyl)-bridged complex **2** was achieved. **2** is composed of a dianionic Bbim^2–^ bridge capped by [{(Me_3_Si)_2_NC(N^i^Pr)_2_}_2_Y]^+^ units and represents the second homonuclear bis(benzimidazolyl)-containing complex bearing RE metals. **2** was reduced chemically to yield a highly soluble and reactive Bbim^•3–^ radical-bridged species, **2′**. Electrochemcial measurements proved that the guanidinate anions in **2′** tremendously impact the redox potential relative to [(Cp*_2_Y)_2_(μ-Bbim^•^)]^−^ but confirm accessibility of the Bbim^4–^ state, albeit more difficult. The radical nature of **2′** was proven by EPR spectroscopy, where the collected cw-EPR spectrum resembles that of the only other known bis(benzimidazolyl) radical reported in the literature. Simulations of these spectra suggest that the spin largely resides on the aromatic ligand; however, it can partially migrate to the metal when a highly reducing radical such as Bbim^3–•^ is employed. The foregoing results pave the way to radical complexes with ancillary guanidinate scaffolds employing paramagnetic RE metals. Provided they are synthetically accessible through an akin path, such materials have ramifications in various spin-based sciences from quantum computing to molecular magnets to spintronics.

## Supporting Information Available

Computational data, magnetic data, spectroscopic data, and detailed crystallographic information for **1** and **2** (PDF)
