# Supplementary material for: Guanidinate Yttrium Complexes Containing Bipyridyl and Bis(benzimidazolyl) Radicals
Source: Inorg Chem. 2024 Apr 3;63(21):9659–69. doi: 10.1021/acs.inorgchem.4c00006 (PMC11134503; doi:10.1021/acs.inorgchem.4c00006)
Supplement: Supplementary file 1 — ic4c00006_si_001.pdf [file ic4c00006_si_001.pdf]

Supporting Information

Guanidinate Yttrium Complexes Containing Bipyridyl and Bis(benzimidazolyl) Radicals

Francis Delano IV,[§] Saroshan Deshapriya,[§] and Selvan Demir*

Department of Chemistry, Michigan State University,
578 South Shaw Lane, East Lansing, Michigan 48824, USA

*Correspondence to: sdemir@chemistry.msu.edu (S.D.)
[§]These authors contributed equally.

Table of Contents

Figure S1. Classes of RE bpy radical complexes S2

X–Ray Crystallography **S4**

Table S1. Crystal data and structure refinement of {$(Me_3Si)_2NC(N^iPr)_2$}$_2$Y(bpy•), **1** and [{$(Me_3Si)_2NC(N^iPr)_2$}$_2$Y]$_2$(μ–Bbim), **2** S5

Figure S2. Structure of {$(Me_3Si)_2NC(N^iPr)_2$}$_2$Y(bpy•), **1** S6

Figure S3. Unit cell of {$(Me_3Si)_2NC(N^iPr)_2$}$_2$Y(bpy•), **1** S6

Figure S4. Space filling model of {$(Me_3Si)_2NC(N^iPr)_2$}$_2$Y(bpy•), **1** S7

Figure S5. Structure of [{$(Me_3Si)_2NC(N^iPr)_2$}$_2$Y]$_2$(μ–Bbim), **2** S7

Figure S6. Space filling model of [{$(Me_3Si)_2NC(N^iPr)_2$}$_2$Y]$_2$(μ–Bbim), **2** S8

Figure S7. Unit cell of [{$(Me_3Si)_2NC(N^iPr)_2$}$_2$Y]$_2$(μ–Bbim), **2** S8

IR Spectroscopy **S9**

Figure S8. FTIR spectrum of {$(Me_3Si)_2NC(N^iPr)_2$}$_2$Y(bpy•), **1** S9

Figure S9. FTIR spectrum of [{$(Me_3Si)_2NC(N^iPr)_2$}$_2$Y]$_2$(μ–Bbim), **2** S9

NMR Spectroscopy **S10**

Figure S10. ^{1}H NMR spectrum of [{$(Me_3Si)_2NC(N^iPr)_2$}$_2$Y]$_2$(μ–Bbim), **2** S10

Figure S11. ^{1}H–^{1}H gCOSY spectrum of [{$(Me_3Si)_2NC(N^iPr)_2$}$_2$Y]$_2$(μ–Bbim), **2** S10

Figure S12. ^{13}C NMR spectrum of [{$(Me_3Si)_2NC(N^iPr)_2$}$_2$Y]$_2$(μ–Bbim), **2** S11

EPR Spectroscopy **S12**

Figure S13. Variable temperature cw-EPR spectra of {$(Me_3Si)_2NC(N^iPr)_2$}$_2$Y(bpy•), **1,** and [K(crypt-222)][{$(Me_3Si)_2NC(NiPr)_2$}$_2$Y]$_2$(μ–Bbim•), **2′** S12

Cyclic Voltammetry **S13**

Figure S14. Cyclic voltammogram of {$(Me_3Si)_2NC(N^iPr)_2$}$_2$Y(bpy•), **1** in difluorobenzene S13

Figure S15. Cyclic voltammogram of [{$(Me_3Si)_2NC(N^iPr)_2$}$_2$Y]$_2$(μ–Bbim), **2** in THF S14

DFT Calculations **S15**

Figure S16. Comparison of experimental FTIR spectrum and calculated stretching frequencies of **1** S15

Figure S17. Frontier orbitals of
[K(crypt-222)][{$(Me_3Si)_2$NC(N^{i}Pr)$_2$}$_2$Y]$_2$(μ–Bbim•), **2′** S16
Table S2. TDDFT–calculated transitions for
{$(Me_3Si)_2$NC(N^{i}Pr)$_2$}$_2$Y(bpy•), **1** S17
Table S3. TDDFT–calculated transitions for
[K(crypt-222)][{$(Me_3Si)_2$NC(N^{i}Pr)$_2$}$_2$Y]$_2$(μ–Bbim•), **2′** S20
Table S4. Comparison of calculated and experimentally
determined hyperfine coupling constants
of {$(Me_3Si)_2$NC(N^{i}Pr)$_2$}$_2$Y(bpy•), **1** S21
Table S5. Vibrational modes for
[K(crypt-222)][{$(Me_3Si)_2$NC(N^{i}Pr)$_2$}$_2$Y]$_2$(μ–Bbim•), **2′** S22
Table S6. Optimized coordinates
of {$(Me_3Si)_2$NC(N^{i}Pr)$_2$}$_2$Y(bpy•), **1** S28
Table S7. Optimized coordinates
of [K(crypt-222)][{$(Me_3Si)_2$NC(N^{i}Pr)$_2$}$_2$Y]$_2$(μ–Bbim•), **2′** S31

References S37

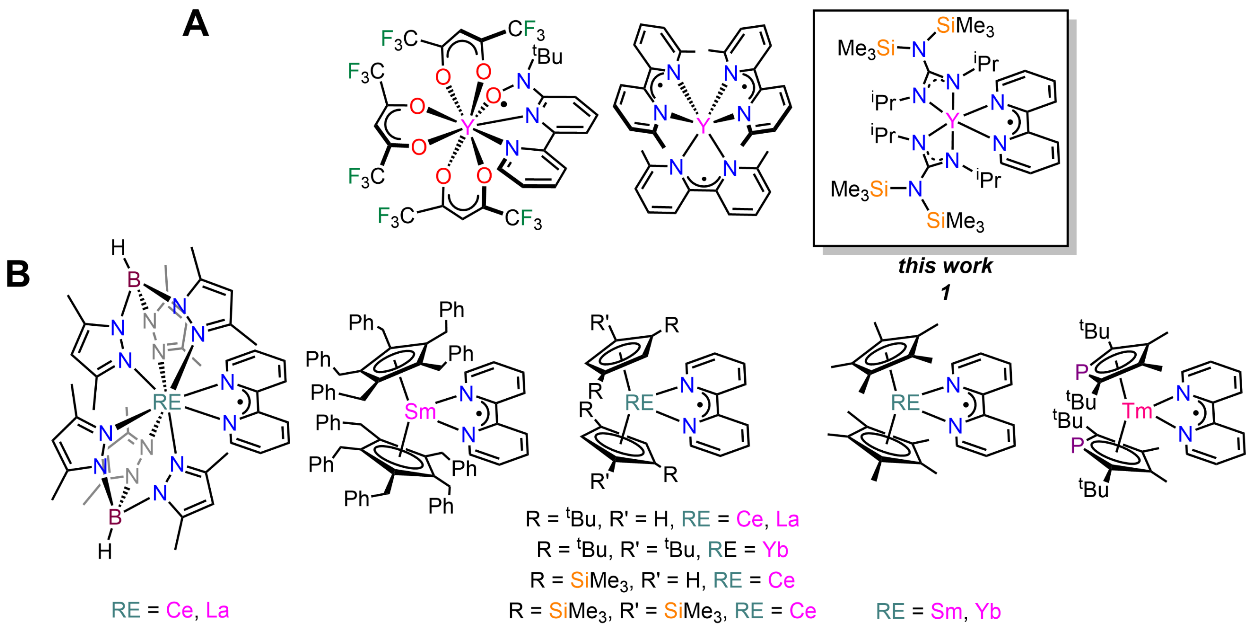

Figure S1. (**A**) Yttrium complexes containing bare bpy radicals, where the first comprises the radical on the NO ligand. (**B**) Classes of RE bpy radical complexes of the formula $L_2RE(bpy•)$ where L = trispyrazolylborates, bulky cyclopentadienyls, and phospholyls, respectively.

X–Ray Crystallography

Table S1. Crystal data and structure refinement of $\{(Me_3Si)_2NC(N^iPr)_2\}_2Y(bpy\bullet)$, **1**, and $[\{(Me_3Si)_2NC(N^iPr)_2\}_2Y]_2(\mu$–Bbim), **2**. **1** crystallized with one *n*–hexane solvent molecule in the lattice, as: $\{(Me_3Si)_2NC(N^iPr)_2\}_2Y(bpy\bullet)\cdot C_6H_{14}$. **2** crystallized with two pentane solvent molecules in the lattice, as: $[\{(Me_3Si)_2NC(N^iPr)_2\}_2Y]_2(\mu$–Bbim)$\cdot 2C_5H_{12}$.

Compound	**1**	**2**
Empirical formula	$C_{42}H_{86}N_8Si_4Y$	$C_{76}H_{160}N_{16}Si_8Y_2$
CCDC Number	2314863	2314864
Formula weight (g mol^{-1})	904.45	1700.73
Temperature (K)	100.0(1)	100.0(1)
Crystal system	Monoclinic	Triclinic
Space group	$P2_1/n$	P-1
Unit Cell Dimensions	a = 10.02584(10) Å	a = 15.12930(10) Å
	b = 31.0536(3) Å	b = 18.7107(2) Å
	c = 16.66609(15) Å	c = 19.8814(2) Å
	α = 90°	α = 65.9560(10)°
	β = 96.7591(9)°	β = 74.7580(10)°
	γ = 90°	γ = 85.4370(10)°
Volume (Å^3)	5152.74(9)	4956.48(9)
Z	4	2
ρ_{calc} (g cm^{-3})	1.166	1.140
μ (mm^{-1})	2.748	2.827
F(000)	1956.0	1836.0
Crystal size (mm^3)	0.614 × 0.119 × 0.074	0.315 × 0.2 × 0.116
Radiation	Cu K_α (λ = 1.54184)	Cu K_α (λ = 1.54184)
2Θ range for data collection (°)	5.692 to 160.732	5.032 to 155.556
Reflections collected	46745	86316
Independent reflections	11009 R_{int} = 0.0419	20640 R_{int} = 0.0438
Data/restraints/parameters	11009/150/575	20640/139/1079
Goodness–of–fit on F^2	1.052	1.061
Final R indexes [I>=2σ (I)]	R_1 = 0.0380, wR_2 = 0.0961	R_1 = 0.0433, wR_2 = 0.1156
Final R indexes [all data]	R_1 = 0.0430, wR_2 = 0.0990	R_1 = 0.0482, wR_2 = 0.1186
Largest diff. peak/hole (e Å^{-3})	0.86/–0.83	2.07/–0.60

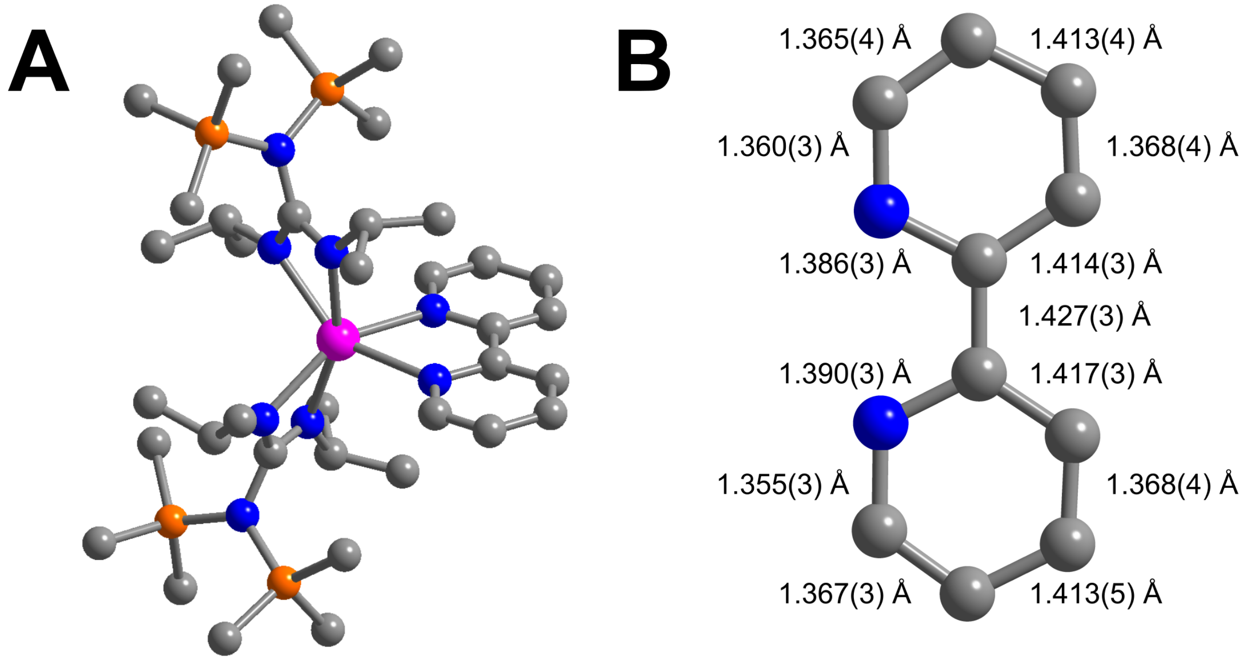

Figure S2. (**A**) Structure of $\{(Me_3Si)_2NC(N^iPr)_2\}_2Y(bpy•)$, **1**, in a crystal of $\{(Me_3Si)_2NC(N^iPr)_2\}_2Y(bpy•)·C_6H_{14}$. Pink, orange, blue, and gray spheres represent Y, Si, N, and C atoms, respectively. Hydrogen atoms and cocrystallized hexane have been omitted for clarity. (**B**) Interatomic C–C and C–N distances of the coordinated bpy anion.

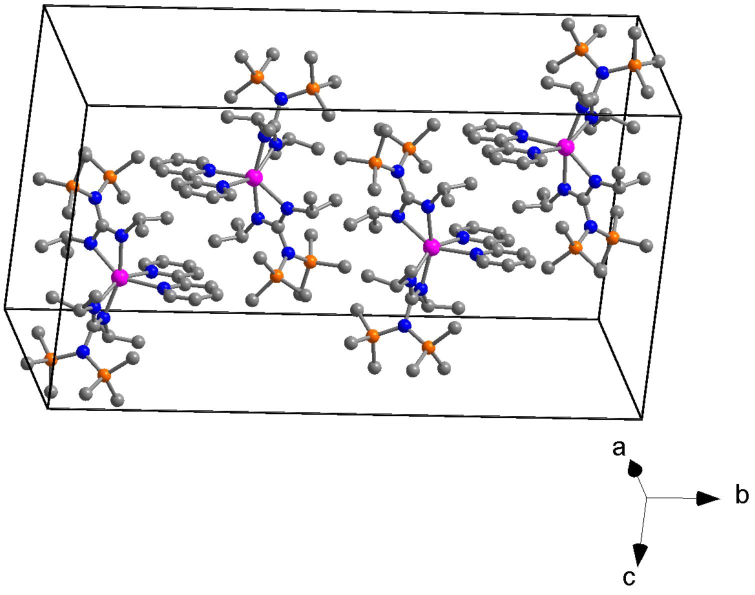

Figure S3. Unit cell of $\{(Me_3Si)_2NC(N^iPr)_2\}_2Y(bpy•)·C_6H_{14}$. Pink, orange, blue, and gray spheres represent Y, Si, N, and C atoms, respectively. Hydrogen atoms and cocrystallized hexane have been omitted for clarity.

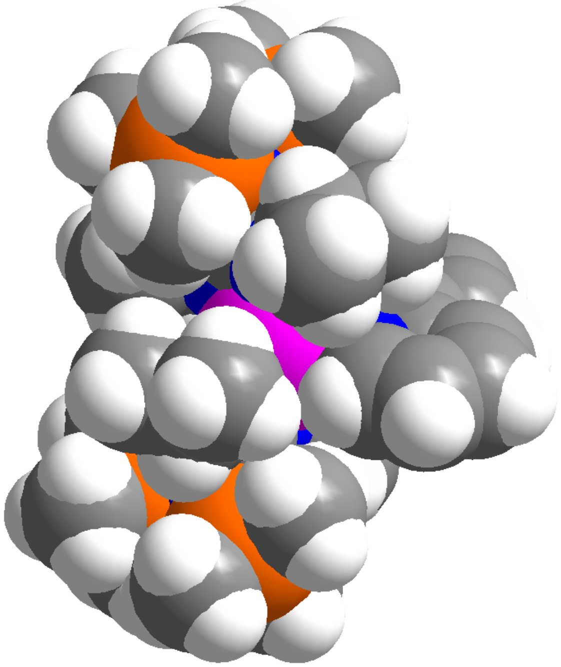

Figure S4. Space filling model of {$(Me_3Si)_2NC(N^iPr)_2$}$_2$Y(bpy•), **1**, in a crystal of {$(Me_3Si)_2NC(N^iPr)_2$}$_2$Y(bpy•)·C_6H_{14}. Pink, orange, blue, gray and white spheres represent Y, Si, N, C, and H atoms, respectively. Cocrystallized hexane has been omitted for clarity.

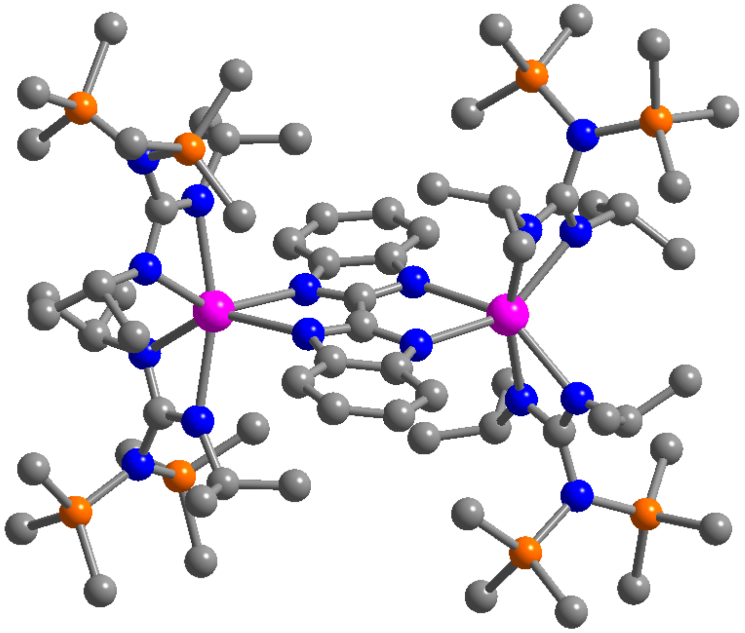

Figure S5. Structure of [{$(Me_3Si)_2NC(N^iPr)_2$}$_2$Y]$_2$(*μ*–Bbim), **2**, in a crystal of [{$(Me_3Si)_2NC(N^iPr)_2$}$_2$Y]$_2$(*μ*–Bbim)·$2C_5H_{12}$. Pink, orange, blue, and gray spheres represent Y, Si, N, and C atoms, respectively. Hydrogen atoms and cocrystallized pentane have been omitted for clarity.

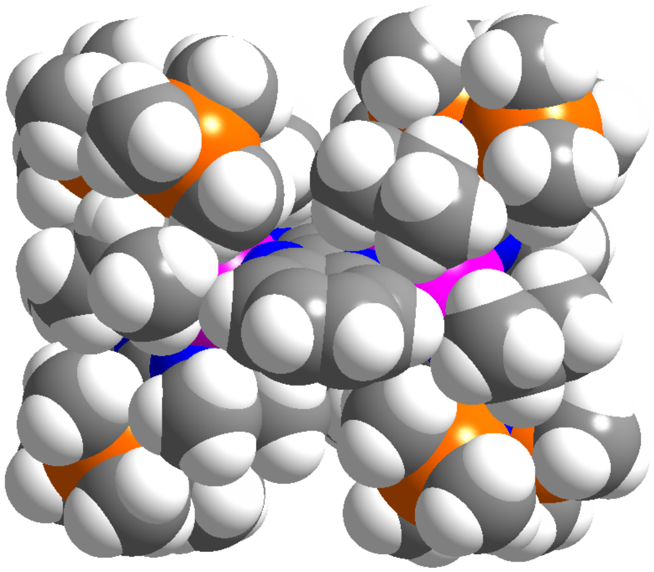

Figure S6. Space filling model of [{$(Me_3Si)_2NC(N^iPr)_2$}$_2Y]_2$(μ–Bbim), **2**, in a crystal of [{$(Me_3Si)_2NC(N^iPr)_2$}$_2Y]_2$(μ–Bbim)·$2C_5H_{12}$. Pink, orange, blue, gray, and white spheres represent Y, Si, N, C, and H atoms, respectively. Cocrystallized pentane has been omitted for clarity.

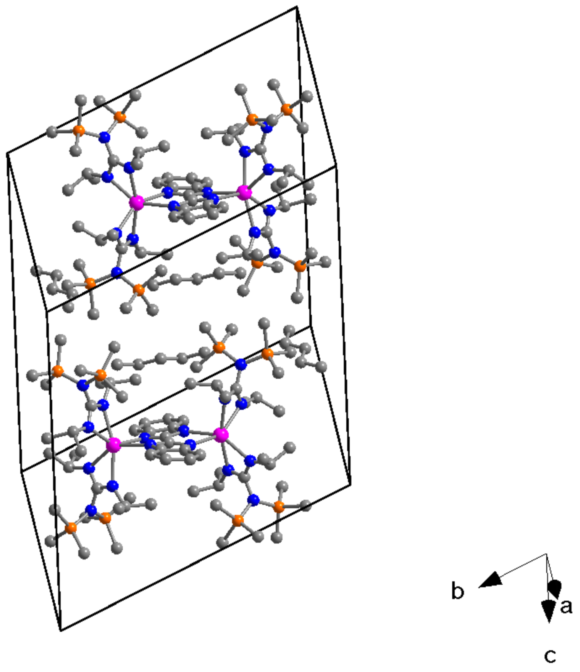

Figure S7. Unit cell of [{$(Me_3Si)_2NC(N^iPr)_2$}$_2Y]_2$(μ–Bbim)·$2C_5H_{12}$. Pink, orange, blue, and gray spheres represent Y, Si, N, and C atoms, respectively. Hydrogen atoms have been omitted for clarity.

IR Spectroscopy

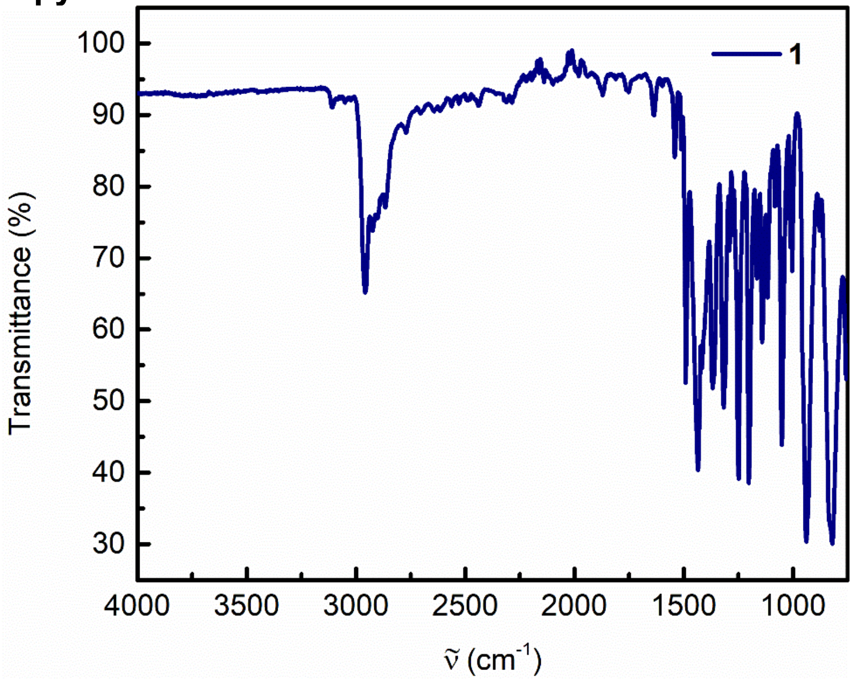

Figure S8. FTIR spectrum of {$(Me_3Si)_2NC(N^iPr)_2\}_2Y(bpy•)$, **1**.

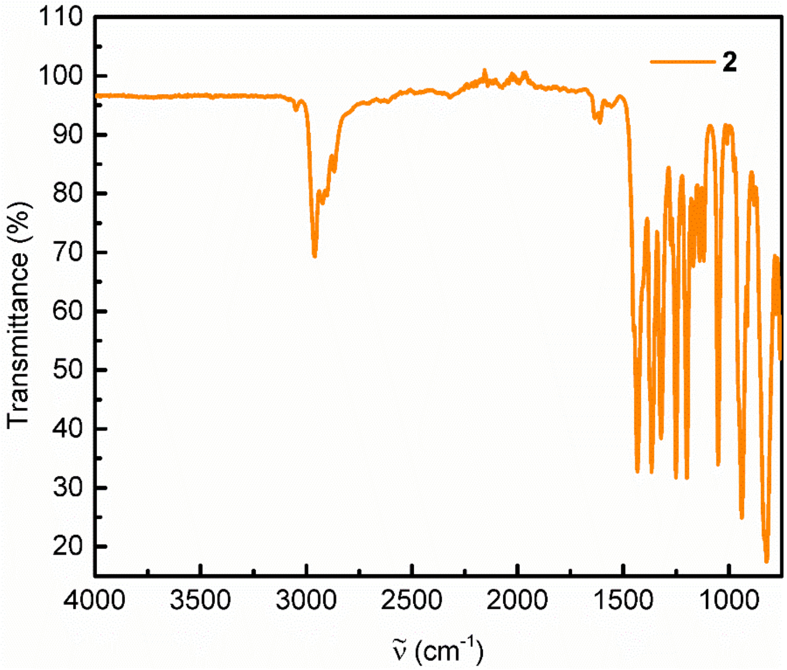

Figure S9. FTIR spectrum of $[\{(Me_3Si)_2NC(N^iPr)_2\}_2Y]_2(\mu\text{–Bbim})$, **2**.

NMR Spectroscopy

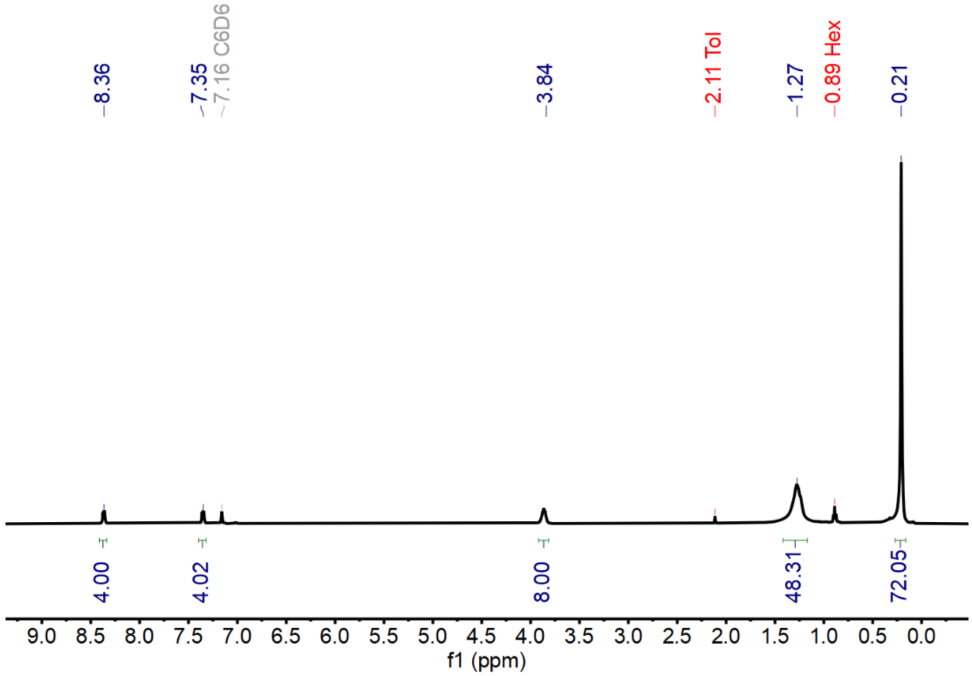

Figure S10. ^{1}H NMR spectrum of [{(Me$_3$Si)$_2$NC(N^{i}Pr)$_2$}$_2$Y]$_2$(μ–Bbim), **2**, (500 MHz, benzene-*d*$_6$, 25 °C) δ 0.21 (s, 72 H, Si(C*H*$_3$)$_3$), 1.27 (br s, 48 H, CH(C*H*$_3$)$_2$), 3.84 (br s, 8 H, C*H*(CH$_3$)$_2$), 7.36 (dt, $^3J_{H–H}$ = 3.40, and 6.81 Hz, 4 H, Bbim), 8.36 (dt, $^3J_{H–H}$ = 3.46, and 7.02 Hz, 4 H, Bbim).

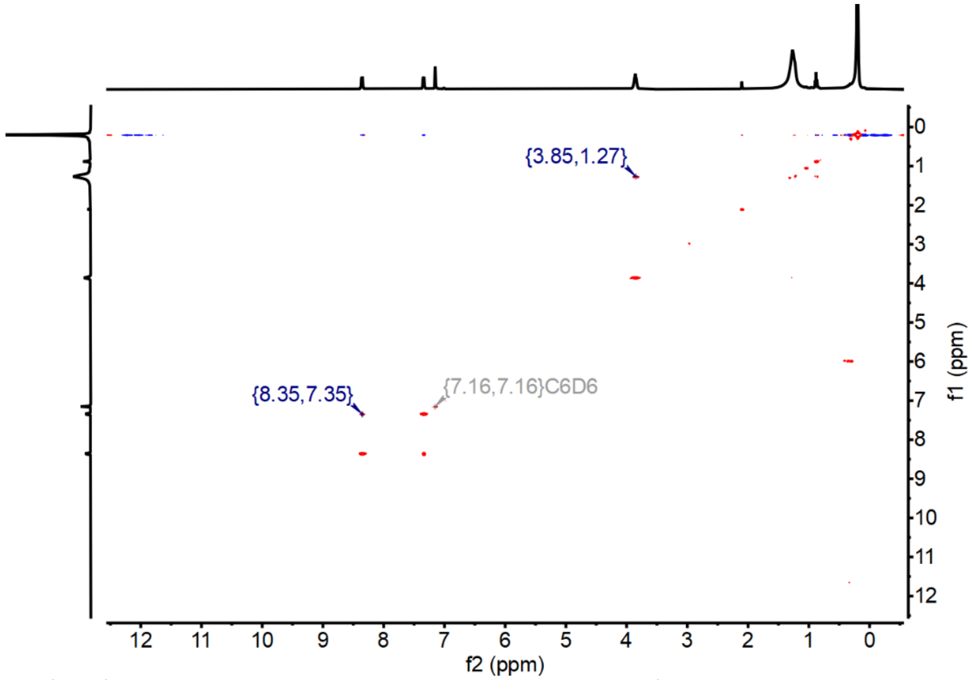

Figure S11. ^{1}H–^{1}H gCOSY spectrum of [{(Me$_3$Si)$_2$NC(N^{i}Pr)$_2$}$_2$Y]$_2$(μ–Bbim), **2**, (500 MHz, benzene-*d*$_6$, 25 °C).

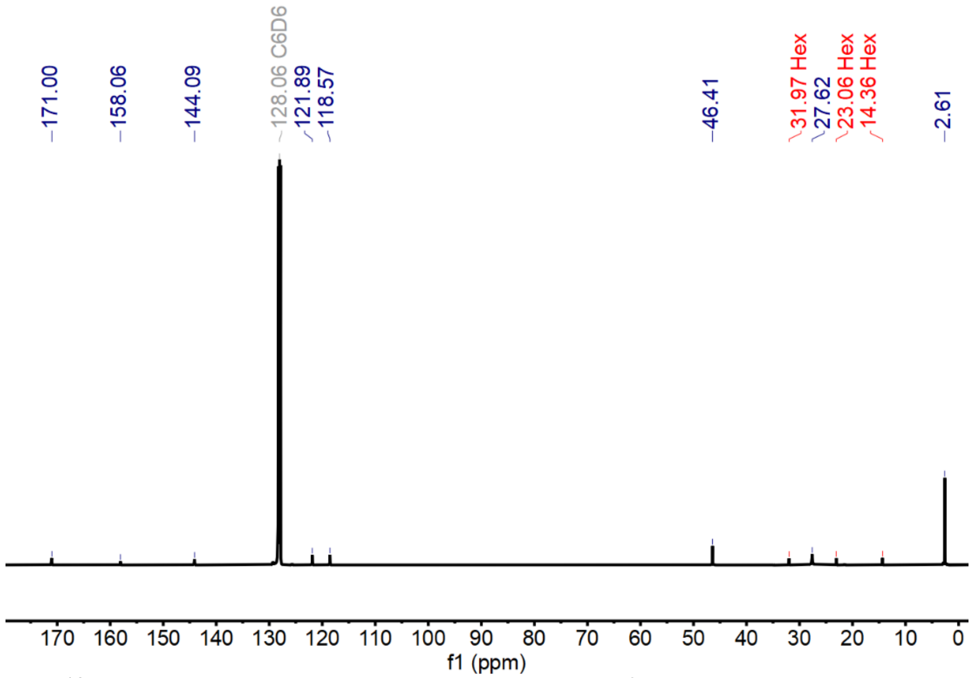

Figure S12. ^{13}C NMR spectrum of $[\{(Me_3Si)_2NC(N^iPr)_2\}_2Y]_2(\mu$–Bbim), **2**, (126 MHz, benzene-d_6, 25 °C) δ 171.0 (<u>*C*</u>N_3), 158.06 (Bbim), 144.09 (Bbim), 121.89 (Bbim), 118.57 (Bbim), 46.41 (<u>*C*</u>$H(CH_3)_2$), 27.62 (CH(<u>*C*</u>$H_3)_2$), 2.61 (Si(<u>*C*</u>$H_3)_3$).

EPR Spectroscopy

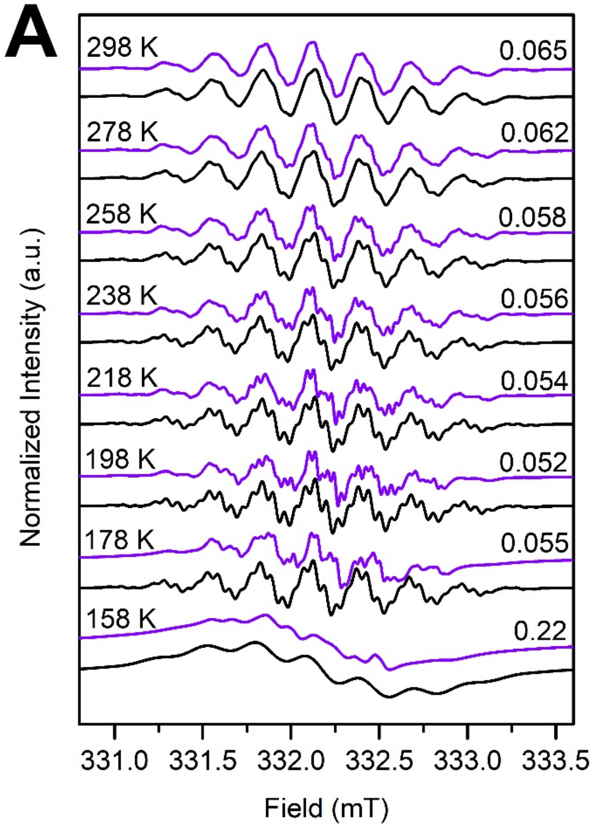

Figure S13. Variable temperature X-band cw-EPR spectra of (**A**) $\{(Me_3Si)_2NC(N^iPr)_2\}_2Y(bpy\bullet)$, **1**, in toluene and (**B**) $[K(crypt\text{-}222)][\{(Me_3Si)_2NC(N^iPr)_2\}_2Y]_2(\mu\text{–}Bbim\bullet)$, **2′**, in THF. Violet and pink lines represent experimental data for **1** and **2′**, whereas black lines represent simulated EPR spectra. Simulation data for **1**: Spin system of 4 ^{14}N, 1 ^{89}Y, 4 ^{1}H and 4 ^{1}H nuclei, $A(^{14}N)$ = 8.31 MHz, $A(^{89}Y)$ = 1.36 MHz, $A_1(^{1}H)$ = 6.90 MHz, $A_2(^{1}H)$ = 1.90 MHz, g = 2.0018. Simulation data for **2′**: Spin system of 4 ^{14}N, 2 ^{89}Y, 4 ^{1}H and 4 ^{1}H. $A(^{14}N)$ = 5.15 MHz, $A(^{89}Y)$ = 0.46 MHz, $A_1(^{1}H)$ = 2.39 MHz, $A_2(^{1}H)$ = 0.54 MHz, g = 2.0032. The values on the right-hand side of the spectra correspond to the linewidth used for the simulation at each temperature.

Cyclic Voltammetry

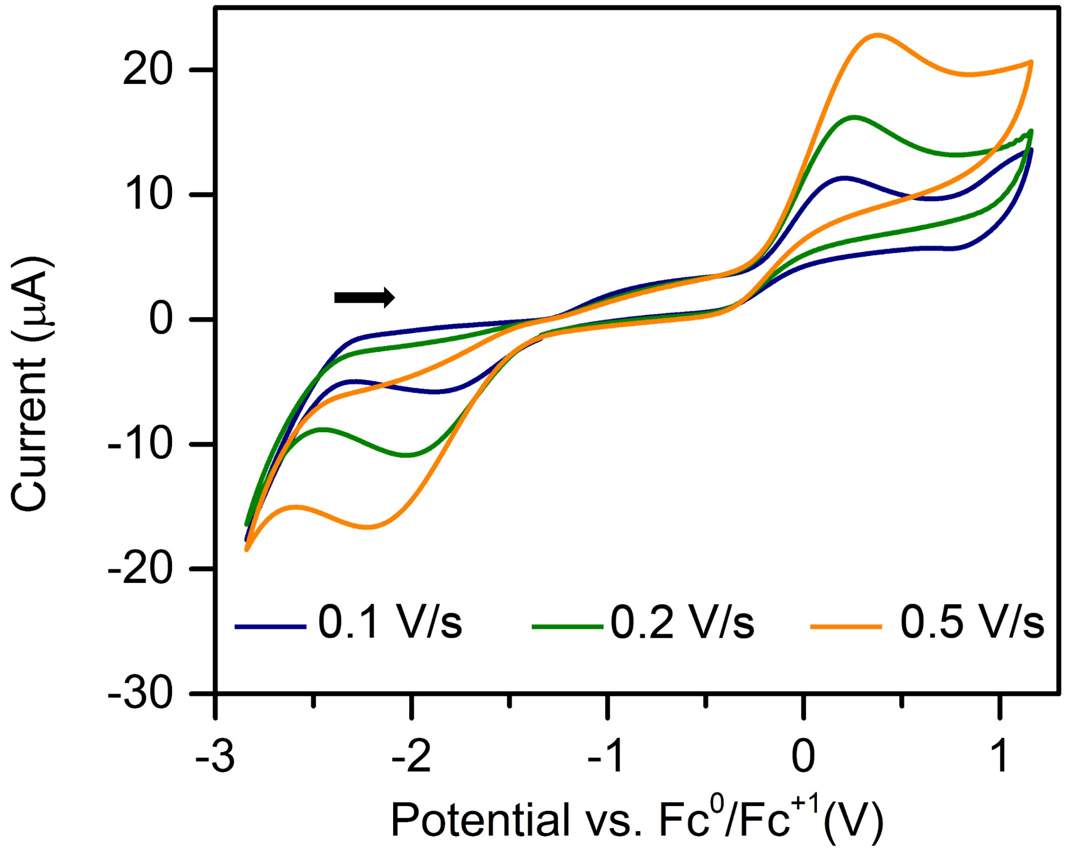

Figure S14. Cyclic Voltammogram of {$(Me_3Si)_2NC(N^iPr)_2$}$_2$Y(bpy•), **1**, vs. Fc. Measurements were taken in 100 mM [^{n}Bu_4N][PF_6] in fluorobenzene with analyte concentration of 2 mM. Measurements were conducted under 0.1 V/s, 0.2 V/s, and 0.5 V/s scan rates which are shown in blue, green, and orange colors respectively. Two quasi-reversible features with half step potentials at –1.81 V and –0.03 V are observed (Fc. redox couple at 1.34 ± 0.03 V).

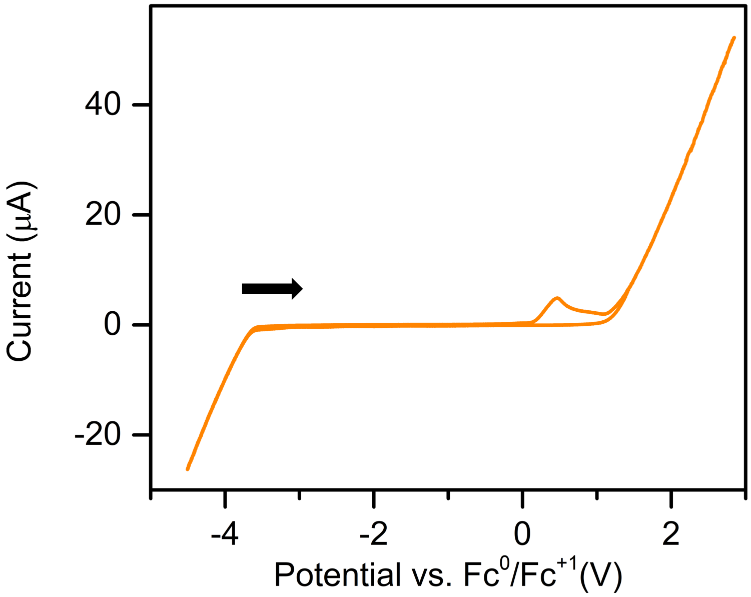

Figure S15. Cyclic Voltammogram of $[\{(Me_3Si)_2NC(N^iPr)_2\}_2Y]_2(\mu\text{–Bbim})$, **2**, vs. Fc. Measurements were taken in 100 mM $[^nBu_4N][BPh_4]$ in THF with analyte concentration of 1 mM. The feature observed around 0.5 V is assigned to the irreversible oxidation of the BPh_4^- ion of the supporting electrolyte.[1]

DFT Calculations

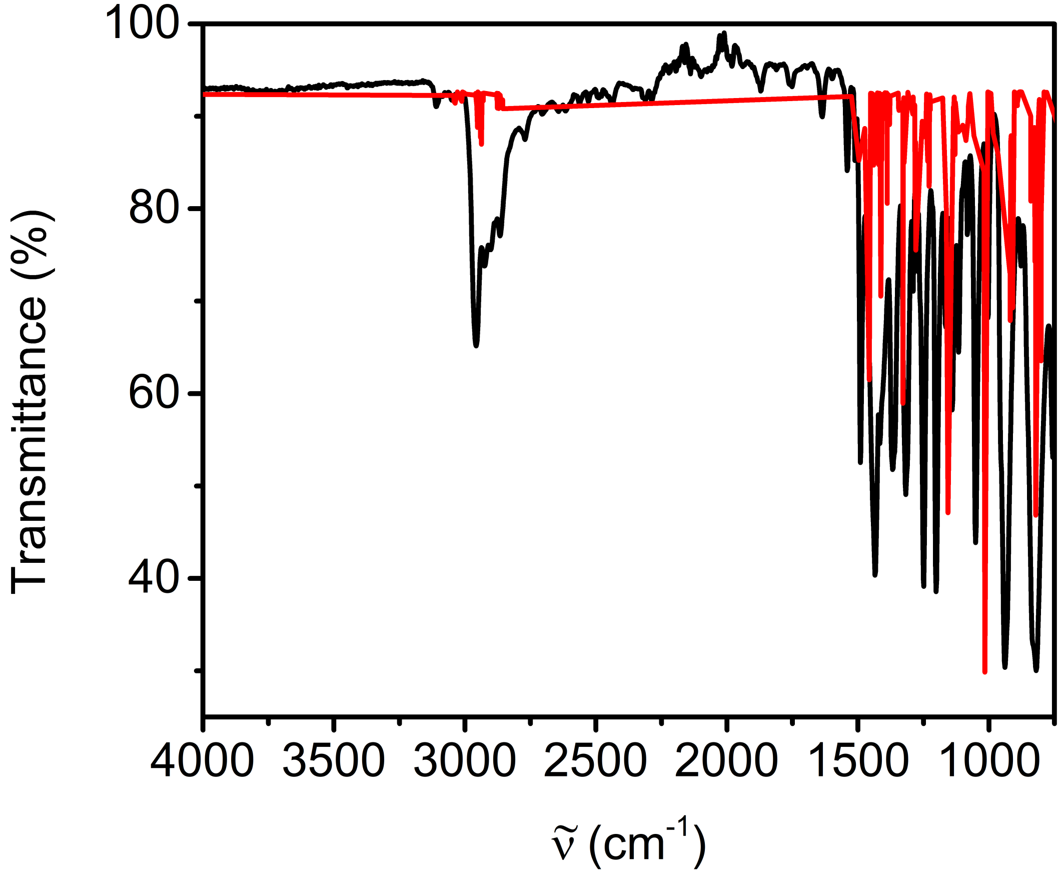

Figure S16. Comparison of experimental FTIR spectrum (black) and calculated stretching frequencies (red) for $\{(Me_3Si)_2NC(N^iPr)_2\}_2Y(bpy\bullet)$, **1.**

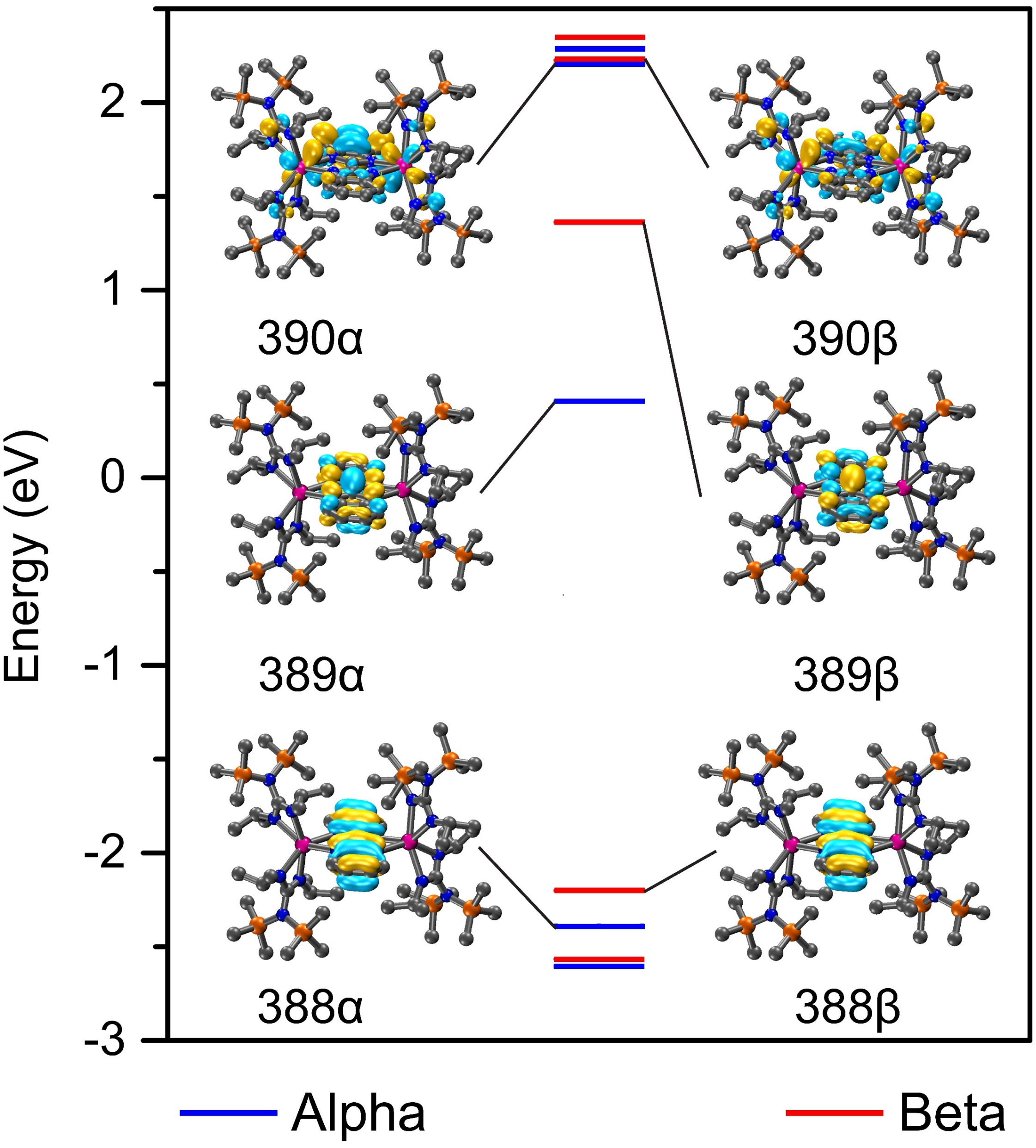

Figure S17. Frontier orbitals of the optimized structure of the $[\{(Me_3Si)_2NC(N^iPr)_2\}_2Y]_2(\mu$–Bbim•$)^-$ anion in $[K(crypt\text{-}222)][\{(Me_3Si)_2NC(N^iPr)_2\}_2Y]_2(\mu$–Bbim•), **2′**. Molecular orbital numbers 388, 389, and 390 correspond to HOMO, SOMO, and LUMO, respectively. Pink, gray, blue, and orange spheres represent Y, C, N, and Si atoms. H atoms have been omitted for clarity. Energy levels are shown to scale.

Table S2. Majority contributions of the TDDFT–calculated transition states for {(Me$_3$Si)$_2$NC(N^{i}Pr)$_2$}$_2$Y(bpy•), **1**, on the def2-TZVP level using the uB3LYP functional with D3BJ dispersion correction and Et$_2$O implicit solvent model. The calculated excitation energies were empirically shifted by 0.3 eV. Isovalue for all depictions is 0.03. Oscillator strength cutoff used is 0.075 and contributions higher than 15% are shown. (HOMO = 204, SOMO = 205, LUMO = 206)

λ (nm)	ν (cm^{-1})	Oscillator Strength	Occupied	Virtual	Weight (%)
368.9	27108	0.31075	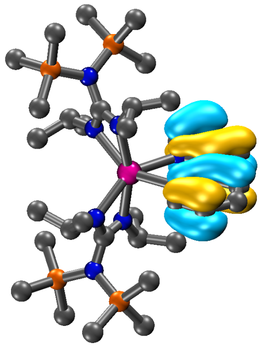 202β	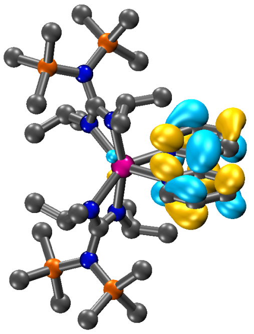 205β	50.6
			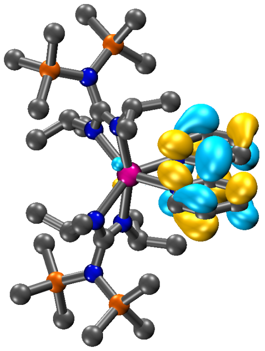 205α	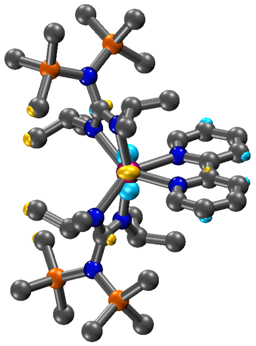 215α	29.3
263.4	37972	0.15537	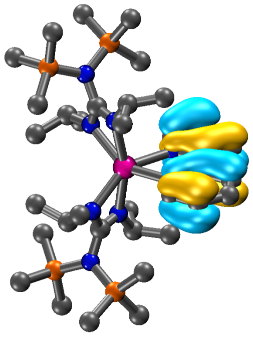 202β	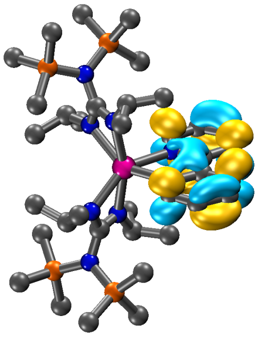 206β	24.8

			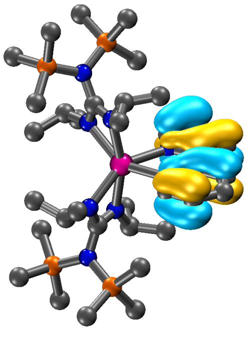 200α	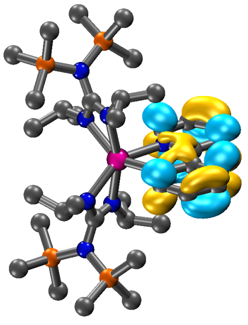 206α	18.4
375.7	26618	0.11285	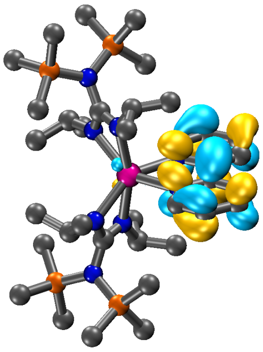 205α	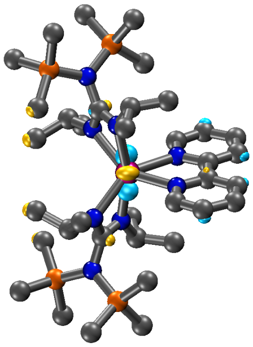 215α	45.1
			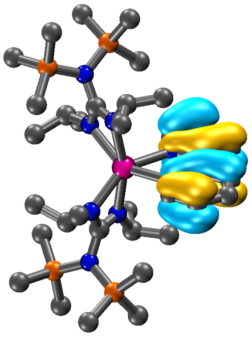 202β	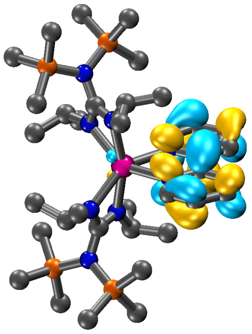 205β	26.4
			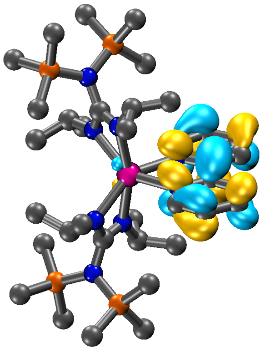 205α	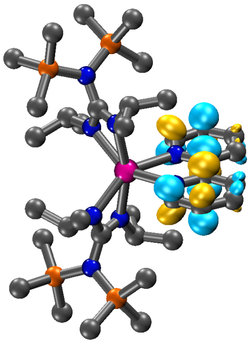 211α	21.9

271.1	36882	0.10102	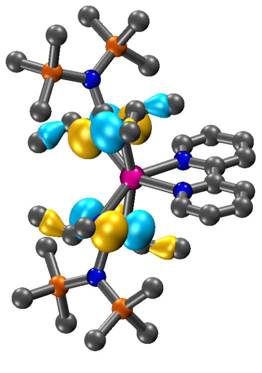 204β	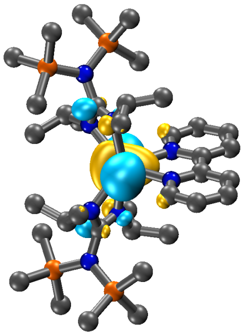 209β	43.9
			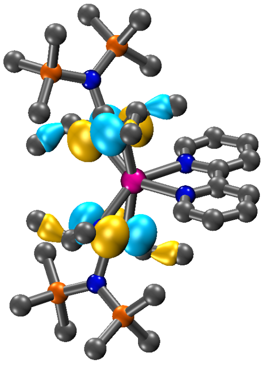 204α	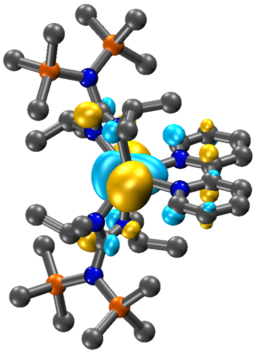 209α	35.7
234.0	42729	0.08230	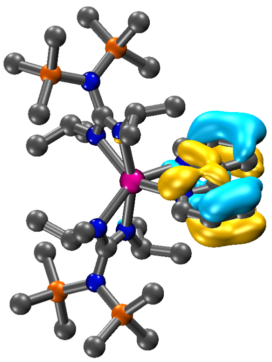 194β	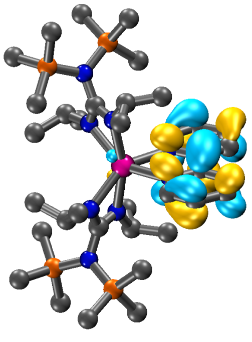 205β	19.9
839.2	11916	0.07556	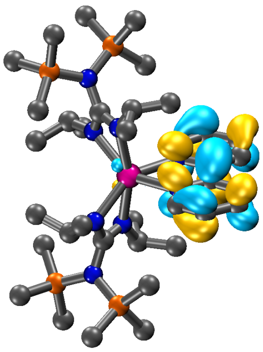 205α	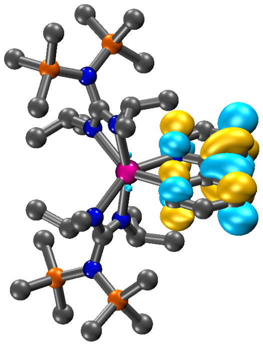 207α	93.3

Table S3. Majority contributions of the TDDFT–calculated transitions of the $[\{(Me_3Si)_2NC(N^iPr)_2\}_2Y]_2(\mu\text{–Bbim}\bullet)^-$ anion in $[K(\text{crypt-222})][\{(Me_3Si)_2NC(N^iPr)_2\}_2Y]_2(\mu\text{–Bbim}\bullet)$, **2′**, on the def2-SVP level using the uB3LYP functional with D3BJ dispersion correction and THF implicit solvent model. The calculated excitation energies were empirically shifted by 0.3 eV. Isovalue for all depictions is 0.03. Oscillator strength cutoff used is 0.05 and contributions higher than 15% are shown. (HOMO = 388, SOMO = 389, LUMO = 390)

λ (nm)	ν (cm^{-1})	Oscillator Strength	Occupied	Virtual	Weight (%)
346.4	28867	0.41421	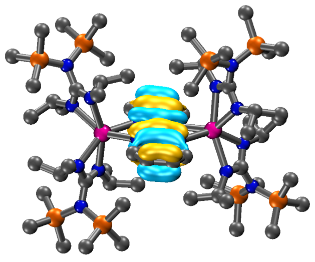 388β	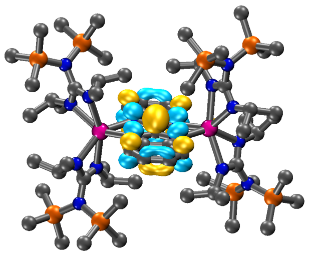 389β	80.7
582.1	17179	0.19158	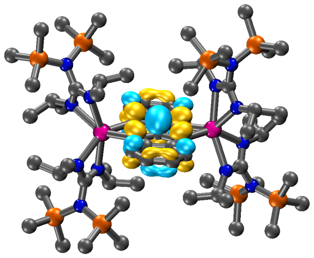 389α	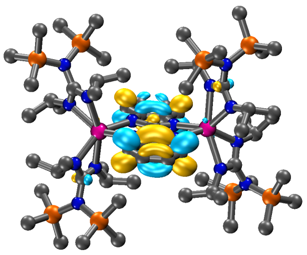 391α	96.3
255.8	39092	0.07448	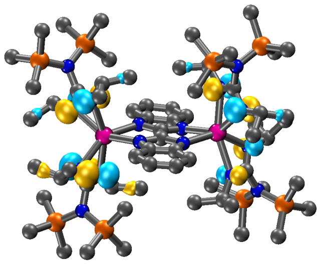 386β	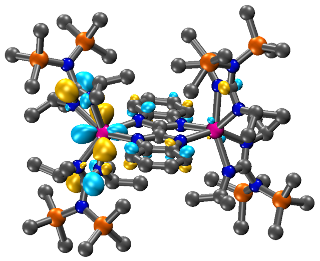 392β	15.3
247.2	40459	0.05372	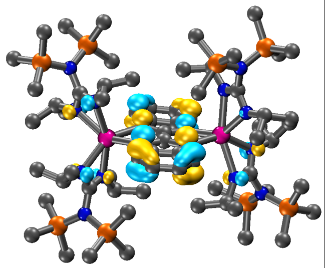 385β	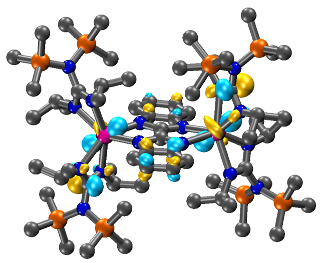 393β	15.8

531.0	18831	0.053027	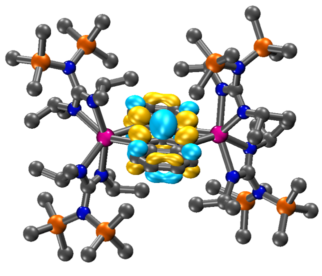 389α	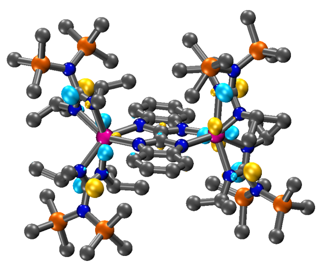 394α	81.1
			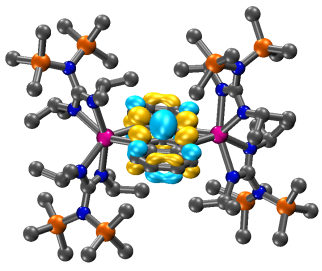 389α	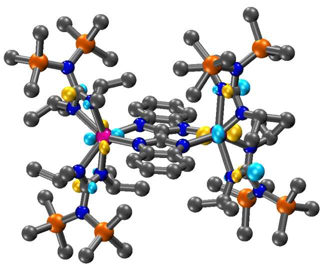 396α	15.9

Table S4. Comparison of DFT calculated hyperfine coupling constants and the hyperfine coupling constants used for simulating cw-EPR spectra of $\{(Me_3Si)_2NC(N^iPr)_2\}_2Y(bpy\bullet)$, **1**. DFT calculations carried out using uTPSSh functional at def2-TZVP level with D3BJ dispersion correction on the optimized structure of **1**.

**Atom**	**DFT calculated hyperfine coupling constants (MHz)**	**Hyperfine coupling constants used for simulation (MHz)**
^{89}Y	0.002728	1.36
^{14}N (Bpy)	13.66598, 13.66351,	8.31
^{1}H (Bpy)	1.91357, 2.39430, 2.39768, 1.90469	6.90
^{1}H (Bpy)	0.06093, 0.05795, 0.06119, 0.05802	1.90

Table S5. Vibrational modes for the optimized structure of **2′** obtained through a frequency calculation on the def2-SVP level using the uTPSSh functional with D3BJ dispersion correction.

Frequency (cm^{-1})	Intensity (a.u.)
13.19	0.03
15.32	0
19.85	0
21.96	0.03
22.87	0
24.69	0.01
27.64	0.04
28.78	0.04
31.00	0.08
32.72	0
35.99	0.05
37.25	0.13
43.47	0.13
44.44	0.08
46.67	0.19
49.43	0.01
50.11	0.03
51.95	0.03
53.41	0.13
55.17	0.18
56.20	0.21
58.63	0.05
59.06	0.05
62.80	0.2
65.26	0.1
66.41	0.06
68.90	0.12
74.93	0.36
76.22	0.18
78.31	0.17
79.37	0.57
82.55	0.45
84.56	0.3
85.29	0.3
86.20	0.2
90.53	0.33
92.63	0.8
93.57	0.37
95.06	0.27
96.33	0.31
98.90	0.58
99.39	0.17
100.50	0.51
101.76	0.31
105.87	0.02
107.99	0.04
108.21	0.11
110.44	0.2
112.39	0.16
114.42	0.32
115.18	0.39
117.08	0.17
117.52	1.24
118.88	0.52
120.50	0.05
123.66	0.18
127.41	0.58
130.19	0.12
130.88	0.15
133.13	0.23
134.40	1.15
135.44	1.10
136.39	1.69
137.77	1.39
139.72	0.45
140.39	0.07
142.14	0.23
143.55	0.54
144.47	0.18
148.93	0.19
149.29	3.04
151.49	0.06
153.08	0.49
153.42	0.07
154.16	0.66
155.25	0.73
155.68	0.7
157.11	0.14
158.28	0.06
159.75	0.35
160.33	0.08
160.55	0.31
161.88	0.06
164.36	1.8
167.48	0.52
168.32	1.06
169.32	1.38
171.93	3.28
172.79	1
175.02	0.3
176.39	0.41
180.05	0.48
181.59	2.24
182.79	2.59
185.70	0.11
186.69	0.49
188.87	1.65
191.36	9.05
196.76	0.25
197.78	15.08
199.66	2.1
200.41	1.43
201.30	0.88
201.93	0.57
202.41	0.11
204.10	2.89
206.46	6.09
206.96	7.32
207.75	0.11
210.33	2.39
210.78	4.46
212.01	0.7
213.01	3.28
215.13	0.54
215.29	0.24
215.34	0.13

216.25	0.24
222.26	1.25
222.55	0.55
222.85	3.75
223.34	2.09
231.17	14.72
233.06	6.65
234.56	0.28
234.78	0.5
235.43	0.85
235.80	4.05
236.43	0.59
237.68	6.04
238.09	4.28
239.06	1.03
239.83	2.93
241.54	1.49
243.01	4.56
243.81	11.83
246.46	25.22
248.13	25.33
249.32	8.64
251.81	8.7
252.83	38.82
256.38	5.56
259.14	3.34
263.95	2.41
267.50	3.39
269.12	4.2
270.60	3.16
271.60	9.75
272.22	3.16
273.33	4.98
273.57	0.66
275.48	1.32
276.77	0.72
279.63	0.59
281.59	0.93
287.31	3.01
290.88	4.5
291.39	3.78
292.69	4.96
297.84	8.14

303.36	3.92
312.85	0.3
316.85	2.77
323.84	0.4
326.53	20.32
328.06	5.12
333.29	52.46
333.86	28.56
351.05	7.73
351.83	10.23
352.56	12.38
353.64	7.09
373.20	1.42
374.38	0.3
377.71	0.89
379.40	0.37
386.00	0.15
414.73	32.03
415.15	73.4
419.55	15.76
423.28	40.64
434.19	14.74
435.76	23.65
437.00	29.9
439.73	10.31
443.52	5.06
449.22	6.13
451.74	5.63
453.46	5.17
460.93	12.51
466.97	7.97
467.25	17.73
467.94	8.76
468.26	20.75
469.62	18.21
470.59	19.85
472.61	35.19
474.21	25
476.02	4.03
484.80	11.01
554.70	0
583.16	0.72
583.33	1.15

583.95	0.48
584.23	1.23
597.64	0
600.58	0.39
603.17	0.44
606.65	2.09
607.78	2.12
608.03	3.62
608.40	2.98
630.67	0.02
643.46	22.75
646.31	1.3
653.20	27.88
654.40	38.7
654.95	11.81
655.38	23.49
669.26	1.69
669.84	1.68
670.60	0.57
670.81	1.29
670.99	8.97
671.55	7.82
671.61	6.19
672.54	3.93
672.62	4.34
672.70	2.76
672.75	0.21
672.87	6.68
675.68	0.1
675.87	0.32
675.99	0.61
677.52	1.82
680.53	15.81
686.63	4.05
687.12	6.18
687.26	4.72
689.41	8.44
689.51	7.19
690.25	6.18
690.62	1.96
690.94	3.02
691.85	8.26
693.45	7.72

693.72	6.62
709.60	19.98
709.80	24.29
710.41	2.45
712.69	17.43
718.53	9.22
751.46	1.82
751.82	1.4
752.01	2.67
752.64	0.32
752.77	2.05
756.23	3.5
756.41	8.86
756.47	4.73
756.60	4.86
767.38	23.52
767.77	23.98
768.17	18.71
768.52	17.87
770.22	14.73
772.41	10.06
772.65	36.83
772.81	91.26
774.17	17.93
775.55	21.8
791.44	3.57
794.66	11.06
795.26	3.23
835.63	1.26
836.93	1.1
837.72	13.5
838.85	2.06
841.66	122.24
841.82	51.33
842.77	264.45
842.92	84.05
846.89	0.93
846.99	6.39
847.45	12.89
847.62	4.53
850.66	29.05
851.08	19.08
851.12	14.32

851.55	15.46
854.24	165.78
854.64	171.42
855.44	20.05
857.61	98.23
861.84	83.66
862.00	193.2
862.64	287.57
863.38	51.6
867.51	3.67
867.76	23.53
868.01	277.36
870.18	57.85
871.26	0.47
877.53	0.47
884.01	0.39
892.52	14.35
892.92	20.19
894.72	74.47
895.49	0.69
919.87	1.25
920.16	1.24
920.74	0.62
923.14	1.36
923.99	0.56
924.50	0.42
925.30	0.22
926.23	1.45
928.82	43.65
930.70	1.84
940.66	1.17
941.48	2.16
942.10	5.82
942.32	3.09
942.34	3.85
942.76	5.62
943.83	5.5
943.94	2.41
959.08	8.03
961.95	175.58
965.49	159.21
966.54	18.93
966.84	103.06

967.56	152.22
973.47	34.63
974.65	48.5
975.37	37.11
976.51	4.48
979.05	145.55
979.33	254.5
980.27	169.13
981.25	51.23
983.89	9.54
1002.52	0
1002.79	0.01
1007.59	0.02
1037.40	12.92
1039.74	0.3
1076.10	84.64
1077.39	602.63
1078.43	148.33
1080.32	16.26
1121.63	0.22
1123.06	2.4
1136.89	5.25
1137.57	5.76
1138.71	0.61
1139.53	1.78
1143.51	23.71
1143.72	21.14
1144.83	10.52
1145.10	15.12
1162.73	0.39
1166.20	0.97
1166.78	19.6
1167.39	38.56
1167.43	31.36
1167.82	15.48
1183.90	2.24
1184.60	4.15
1185.76	4.21
1186.64	15.19
1191.92	45.52
1192.35	29.63
1193.18	3.19
1193.72	8.7

1211.66	2.32
1231.56	202.65
1232.70	644.69
1236.27	431.6
1239.66	5.28
1243.40	3.17
1245.36	14.91
1276.01	7.31
1276.23	1.76
1276.51	0.42
1276.59	14.86
1276.64	13.9
1276.83	16.64
1276.94	0.3
1276.95	1.32
1278.50	59.5
1278.70	68.81
1278.98	23.37
1279.63	1.05
1280.50	51.92
1282.22	23.27
1282.43	56.81
1283.19	77.49
1285.41	54.18
1287.56	134.98
1287.74	41.61
1288.11	21.61
1289.47	31.04
1291.43	182.4
1292.84	22.19
1293.66	10.64
1294.31	1.24
1306.68	24.43
1307.97	7.33
1316.57	1.95
1325.98	24.13
1343.25	40.58
1346.42	82.22
1347.53	29.99
1347.60	22.12
1349.23	54.39
1349.74	45.62
1351.72	18.68

1352.64	5.94
1352.70	28.03
1353.59	21.87
1354.63	53.89
1355.16	26.07
1355.42	23.09
1356.69	35.16
1359.27	14.92
1360.71	18.44
1371.85	0.34
1388.69	2.21
1389.68	10.31
1390.07	15.36
1391.11	1.92
1391.65	16.57
1391.87	0.65
1392.03	8.4
1393.05	5.63
1401.11	0.12
1403.32	23.17
1403.65	25.19
1403.84	25.55
1404.91	8.46
1405.37	10.43
1406.86	2.01
1407.01	1.27
1407.46	0.34
1410.21	54.93
1429.98	156.4
1431.58	211.01
1433.08	149.92
1436.06	162.47
1440.01	18.27
1440.09	5.12
1440.67	2.33
1440.86	6.47
1442.32	0.97
1442.57	29.23
1442.96	9.3
1443.50	32.45
1447.44	16.93
1447.67	9.68
1447.87	2.46

1448.18	6.64
1448.33	4.12
1448.47	0.75
1448.51	6.36
1449.04	2.06
1449.52	5.07
1449.71	0.97
1449.78	4.06
1450.96	1.1
1451.22	3.88
1451.43	4.86
1451.62	6.03
1452.92	9.75
1453.10	3.6
1453.23	1.32
1453.42	0.64
1454.11	0.96
1454.84	1.51
1455.24	6.06
1455.69	0.86
1455.96	9.81
1457.96	0.52
1458.48	0.19
1458.54	0.13
1459.33	0.72
1462.46	1.17
1463.52	0.54
1463.62	4.13
1463.84	2
1463.93	4.37
1466.32	0.69
1466.82	21.55
1466.94	3.16
1467.71	5.58
1468.49	0.76
1468.90	2.85
1469.09	7.76
1469.53	0.39
1469.85	0.22
1473.29	3.76
1474.21	2.72
1474.33	1.85
1475.42	4.56

1475.53	5.71
1475.72	4.43
1476.61	4.67
1477.39	10.31
1477.79	1.5
1480.97	7
1483.25	3.13
1484.83	2.03
1485.82	0.74
1486.29	0.06
1486.54	1.64
1486.96	4.54
1487.12	0.74
1487.74	0.34
1487.89	1.99
1488.81	2.8
1489.16	2.06
1489.83	1.39
1490.39	1.68
1491.16	0.21
1498.06	4.26
1499.02	10.06
1501.42	5.85
1502.47	11.64
1502.59	0.93
1504.23	8.14
1506.44	4.8
1507.35	31.28
1508.75	16.84
1509.28	8.28
1543.65	575.23
1544.98	247
1549.78	292.12
1550.59	923.57
1612.56	0.01
1625.40	22.03
1649.77	4.43
1650.74	25.82
1689.26	0.11
3002.50	34.51
3005.34	35.61
3006.67	29.79
3012.47	27.8

3014.50	33.37
3016.11	24.35
3018.44	24.09
3021.40	9.09
3021.55	8.55
3021.67	7.42
3022.04	14.92
3022.18	17.74
3022.95	11.8
3023.21	9.34
3023.24	5.47
3023.32	7.21
3023.56	24.69
3024.36	9.51
3025.41	25.21
3025.51	45.72
3027.36	4.39
3027.76	65.19
3028.36	13.22
3028.45	21.81
3028.72	10.05
3028.87	12.02
3029.22	4.06
3029.73	19.93
3030.14	24.67
3030.46	53.49
3030.53	18.69
3030.92	9.76
3031.12	17.8
3031.34	8.5
3032.47	10.23
3033.00	24.06
3033.39	9.57
3034.76	11.19
3035.07	9.15
3035.55	15.42
3035.59	43.01
3035.84	6.05
3037.36	25.72
3038.77	26.1
3040.68	4.04
3041.86	4.52
3042.99	3.23

3044.30	2.84
3104.25	22.13
3107.21	10.26
3107.58	15.86
3107.63	16.38
3108.00	18.91
3108.41	15.81
3108.57	29.46
3108.93	7.01
3109.13	8.51
3109.14	16.73
3109.25	12.74
3110.94	24.91
3111.04	29.27
3112.19	27.45
3112.91	54.47
3113.29	35.02
3113.75	50.97
3114.31	17.97
3114.76	10.78
3115.10	13.73
3115.56	8
3115.72	11.8
3116.19	45.72
3116.80	49.48
3117.09	5.68
3117.59	4.36
3117.91	5.12
3119.25	4.51
3119.80	9.87
3120.11	9.44
3120.67	10.29
3120.97	11.64
3122.33	2.61
3123.11	14.39
3123.32	22.56
3123.57	2.76
3123.62	7.99
3124.33	15.31
3124.48	4.11
3124.61	27.52
3124.68	17.97
3125.28	10.97

3125.50	20.17
3126.81	8.13
3127.50	15.34
3128.64	12.68
3129.03	7.96
3129.14	14.67
3129.85	22.57
3129.89	28.44
3130.90	7.77
3131.56	25.53
3132.88	34.13
3133.37	20.38
3135.03	14.08
3135.44	40.27
3136.70	18.41
3137.98	6.73

3138.62	26.68
3139.08	30.96
3139.36	2.82
3139.49	3.16
3140.77	4.95
3141.95	5.82
3142.57	4.53
3143.01	7.91
3143.12	15.26
3144.11	6.25
3144.13	22.15
3145.58	3.24
3145.59	9.07
3145.75	11.31
3146.48	4.44
3146.60	5.46

3147.53	0.95
3148.06	3.72
3148.46	8.02
3151.55	11.05
3154.30	4.05
3159.28	7.18
3168.11	4.75
3168.45	5.66
3184.89	22.56
3185.18	18.99
3225.03	3.4
3226.32	0.06
3227.98	1.92
3228.95	1.73

Table S6. Coordinates of the geometry optimized structure of $\{(Me_3Si)_2NC(N^iPr)_2\}_2Y(bpy\bullet)$, **1**, on the def2-TZVP level using the uTPSSh functional with D3BJ dispersion correction.

Y	5.295886	20.805354	10.962875
N	5.135017	20.574040	13.321921
N	6.663326	22.757660	11.158765
N	4.056592	22.817238	10.591803
N	6.858123	19.319060	10.040071
N	3.634824	19.524369	12.012506
N	5.438725	20.356863	8.634684
C	4.014004	19.861322	13.251064
C	5.592471	21.191165	14.557070
C	6.095795	24.010756	10.980250
C	7.975738	22.684721	11.465894
C	4.703109	24.042202	10.668460
C	2.741958	22.803132	10.286053
C	6.505277	19.574057	8.774406
C	7.851015	18.317496	10.389416
C	2.574949	18.564694	11.753277
C	5.025179	20.893688	7.347705
N	3.251773	19.500985	14.405748
C	5.331367	22.701319	14.531699
C	7.086968	20.929038	14.746150
C	6.901227	25.164747	11.118141
C	8.798042	23.773771	11.616609
C	3.972611	25.229958	10.433990
C	1.990676	23.926302	10.042983
N	7.239309	19.060706	7.660123
C	7.159270	17.047068	10.894964
C	8.787120	18.857416	11.470203
C	1.673897	19.065209	10.624883
C	3.177081	17.209194	11.368259
C	3.517096	20.713706	7.170463
C	5.385411	22.379988	7.244796
Si	1.944792	20.576982	14.895012
Si	3.664058	18.025092	15.268651
C	8.232229	25.058520	11.432736
C	2.637166	25.183234	10.123857
Si	6.725154	17.544146	6.932809
Si	8.616822	19.996459	7.083680
C	2.366004	21.563664	16.438286
C	0.403257	19.571115	15.275113
C	1.582025	21.762633	13.494311
C	3.666126	18.339319	17.120938
C	5.372196	17.473508	14.736971
C	2.439680	16.636391	14.940783

C	4.984741	17.161290	7.507073
C	7.851502	16.109839	7.390159
C	6.741438	17.689496	5.059844
C	9.058849	21.275471	8.374909
C	10.085605	18.857147	6.805728
C	8.264339	20.867073	5.455673
H	5.049783	20.754464	15.403055
H	5.885153	23.163803	13.712351
H	5.650135	23.167101	15.468992
H	4.272145	22.915803	14.382553
H	1.968634	18.435360	12.656575
H	2.531451	20.921257	17.305577
H	1.532234	22.232445	16.678061
H	3.255930	22.181039	16.303385
H	8.444601	18.065285	9.503781
H	0.103011	18.955029	14.423461
H	-0.425203	20.248245	15.507635
H	0.534288	18.913594	16.138757
H	1.172817	19.994424	10.901916
H	0.907019	18.324576	10.383714
H	2.263755	19.246630	9.721750
H	6.459464	26.142558	10.977925
H	2.479664	22.261653	13.123836
H	0.890648	22.537407	13.839732
H	1.116613	21.253271	12.648846
H	8.840529	25.948698	11.539124
H	8.366757	21.680309	11.586726
H	3.771365	17.314657	10.457825
H	2.395015	16.466491	11.184415
H	3.834655	16.833525	12.153258
H	9.842809	23.640247	11.862697
H	6.574947	17.275656	11.789088
H	7.889679	16.273419	11.149886
H	6.476389	16.645786	10.145040
H	8.196136	21.864158	8.693019
H	9.801053	21.968415	7.966887
H	9.488105	20.813131	9.265307
H	5.539513	20.351073	6.546536
H	7.305283	19.860019	14.736128
H	7.434249	21.345768	15.695434
H	7.657339	21.402051	13.943170
H	4.876652	17.277833	8.587043
H	4.728924	16.128043	7.253605
H	4.253557	17.816103	7.030004
H	9.348241	19.721978	11.110986
H	9.503559	18.092326	11.780329

H	8.213549	19.159718	12.351142
H	2.287286	21.819284	10.247244
H	3.230500	19.665436	7.267807
H	3.199016	21.071215	6.187396
H	2.976953	21.287393	7.927438
H	10.341525	18.300880	7.711338
H	10.959174	19.452075	6.519646
H	9.910383	18.135238	6.003509
H	4.475945	26.185867	10.495798
H	4.861972	22.947514	8.016717
H	5.100051	22.782595	6.268308
H	6.456258	22.536242	7.382940
H	4.342735	19.153755	17.392013
H	4.003794	17.435903	17.639463
H	2.672523	18.580729	17.507576
H	0.939265	23.839770	9.804299
H	5.481757	17.471194	13.650920
H	5.562406	16.457417	15.095723
H	6.146406	18.125472	15.145039
H	1.425381	16.907586	15.241800
H	2.729348	15.751931	15.518502
H	2.410014	16.347981	13.888446
H	2.086562	26.098598	9.942995
H	8.044490	20.162820	4.650697
H	9.146219	21.444084	5.156814
H	7.426010	21.561887	5.531985
H	8.883118	16.285581	7.077308
H	7.505064	15.200363	6.887415
H	7.856898	15.911840	8.463570
H	6.122955	18.521465	4.713638
H	6.340453	16.768867	4.623165
H	7.748772	17.825066	4.657381

Table S7. Coordinates of the geometry optimized structure of the $[\{(Me_3Si)_2NC(N^iPr)_2\}_2Y]_2(\mu\text{–Bbim•})^-$ anion in $[K(crypt\text{-}222)][\{(Me_3Si)_2NC(N^iPr)_2\}_2Y]_2(\mu\text{–Bbim•})$, **2′**, on the def2-SVP level using the uTPSSh functional with D3BJ dispersion correction. Structure of **2** was used as the initial coordinates for the optimization with the charge and spin multiplicity set to –1 and 1 respectively, omitting the counterion $[K(crypt\text{-}222)]^+$.

Y	5.901677	12.793575	3.939790
N	4.582466	11.260161	5.206294
N	7.234124	10.828070	4.095281
N	7.525614	14.530903	4.012392
N	4.220331	13.874830	2.658412
N	6.336292	14.120237	5.887112
N	5.459570	12.283172	1.643003
C	5.220939	10.033105	5.235134
C	3.377675	11.024130	5.843509
C	8.370907	10.183301	3.641413
C	6.481766	9.816265	4.664818
C	7.378237	14.731113	5.328082
C	8.439763	15.311747	3.200762
C	4.379034	13.059764	1.606899
C	3.229341	14.933398	2.683569
C	6.129395	14.063284	7.324532
C	5.726182	11.252181	0.652476
N	4.498871	9.026557	5.849072
C	3.317467	9.640284	6.219925
C	2.308077	11.881835	6.135241
C	9.501945	10.675647	2.976066
C	8.264278	8.785821	3.950656
N	7.067774	8.564897	4.606054
N	8.312903	15.514080	6.089316
C	7.670942	16.387976	2.419317
C	9.208999	14.413830	2.228993
N	3.417559	12.996407	0.540737
C	2.477157	14.946971	4.016108
C	3.900457	16.294933	2.448101
C	4.668004	14.376401	7.660065
C	6.506739	12.677548	7.861654
C	7.177439	11.349307	0.170852
C	5.452766	9.854818	1.223294
Y	5.837509	7.057786	5.966409
C	2.175181	9.125786	6.848068
C	1.175026	11.353409	6.779798
C	10.518868	9.773130	2.617367
C	9.284695	7.897606	3.582615
Si	9.758194	14.666592	6.691237
Si	7.952032	17.220669	6.377917

Si	2.052583	11.872922	0.768168
Si	3.665923	14.005294	-0.888852
N	7.430607	7.396801	7.724998
N	6.370253	4.955481	4.994032
N	4.352095	5.849480	4.519828
N	5.458528	6.417408	8.223347
C	1.106237	9.997404	7.123527
C	10.411064	8.407836	2.913218
C	9.908289	13.009208	5.825806
C	11.290574	15.715987	6.326969
C	9.705329	14.398070	8.563135
C	8.994635	18.373735	5.297683
C	8.318578	17.673519	8.176736
C	6.131233	17.529501	6.018926
C	2.013745	11.307623	2.554269
C	0.440582	12.767395	0.338535
C	2.166939	10.364541	-0.367826
C	3.314863	13.012845	-2.459313
C	5.452472	14.595171	-0.914060
C	2.520498	15.512464	-0.927489
C	6.565063	7.029842	8.667511
C	8.635538	8.159046	8.009546
C	5.247098	4.895133	4.269293
C	7.326879	3.866757	5.066736
C	3.177043	6.071547	3.691965
C	4.527976	5.753092	9.115416
N	6.765638	7.325306	10.060443
C	8.476621	9.628278	7.600198
C	9.826664	7.531661	7.277236
N	5.043822	3.886374	3.268475
C	8.717835	4.323578	4.613028
C	7.403160	3.333032	6.503132
C	3.231128	7.451033	3.024687
C	1.904498	5.940041	4.538350
C	3.081936	6.073225	8.731867
C	4.754590	4.233403	9.083071
Si	6.045962	8.843207	10.661533
Si	7.679693	6.200733	11.072060
Si	5.885830	4.116667	1.716826
Si	3.954180	2.540514	3.637706
C	7.354214	10.146882	11.071215
C	5.102495	8.493319	12.265383
C	4.867239	9.520101	9.373292
C	8.678821	5.048103	9.972846
C	8.861046	7.148761	12.204138
C	6.568724	5.155343	12.193848

C	7.316375	2.903302	1.468736
C	6.520737	5.879368	1.621044
C	4.656446	3.813381	0.311073
C	3.716789	2.469287	5.502890
C	2.259465	2.660714	2.805495
C	4.743908	0.926664	3.045871
H	11.404954	10.146449	2.096341
H	9.586806	11.734943	2.739793
H	9.170948	15.819758	3.856117
H	5.057060	11.407952	-0.214585
H	6.772961	14.817985	7.814428
H	2.494173	14.767283	1.875253
H	8.831342	8.126272	9.098013
H	2.117600	8.071618	7.115378
H	2.351807	12.935227	5.863856
H	7.870826	11.157037	1.002927
H	7.394471	12.348217	-0.237178
H	7.376996	10.601233	-0.614005
H	4.005893	13.607159	7.236599
H	4.366543	15.353121	7.252179
H	4.514579	14.391879	8.751737
H	9.203462	6.835951	3.809470
H	8.353780	17.008126	1.814064
H	7.113794	17.048144	3.099560
H	6.941346	15.912789	1.746525
H	3.148165	5.306541	2.895276
H	2.997133	10.969581	2.914143
H	1.313315	10.464163	2.668385
H	1.684671	12.106452	3.233796
H	0.334161	12.014266	7.008601
H	5.592773	9.080303	0.451044
H	4.426209	9.782078	1.605781
H	6.134462	9.637048	2.057179
H	8.967443	12.437765	5.821130
H	10.669661	12.391979	6.330747
H	10.217217	13.128096	4.777700
H	6.996038	3.045582	4.404461
H	2.349774	7.611656	2.381587
H	4.132622	7.546481	2.405455
H	3.262559	8.244587	3.784923
H	11.213980	7.725033	2.621413
H	11.380969	15.927446	5.249134
H	12.198093	15.174241	6.644201
H	11.277394	16.678579	6.864652
H	6.362236	12.615772	8.952938
H	7.556098	12.443359	7.638632

H	5.883036	11.910535	7.379832
H	4.637615	2.140465	6.008071
H	2.911232	1.762563	5.762535
H	3.449292	3.459377	5.902852
H	8.510384	13.822183	1.616738
H	9.860613	13.715704	2.772119
H	9.838898	15.015025	1.553345
H	1.906662	14.018678	4.152501
H	1.774567	15.795133	4.062283
H	3.185696	15.039921	4.855011
H	4.631049	16.494806	3.246419
H	3.158421	17.111356	2.440521
H	4.442217	16.309238	1.491370
H	9.685038	15.346935	9.120739
H	10.608444	13.845004	8.875420
H	8.829954	13.805580	8.867781
H	10.072769	18.214286	5.455048
H	8.770021	19.425306	5.548723
H	8.786304	18.231954	4.226474
H	3.931414	12.100945	-2.503850
H	3.549101	13.627896	-3.345262
H	2.256638	12.714332	-2.536468
H	0.311000	13.672164	0.954357
H	-0.418301	12.101246	0.528959
H	0.396769	13.066029	-0.722111
H	8.093780	3.017427	2.238520
H	7.786725	3.090921	0.487645
H	6.976358	1.856208	1.481875
H	0.210716	9.606883	7.615077
H	5.753205	15.020441	0.055525
H	5.584327	15.371992	-1.685852
H	6.140014	13.766065	-1.137400
H	9.085656	5.127079	5.269938
H	8.692751	4.712285	3.585169
H	9.439311	3.490654	4.652061
H	2.103939	10.634618	-1.433054
H	1.329126	9.681062	-0.144413
H	3.101878	9.806633	-0.211442
H	1.882632	6.721758	5.312208
H	1.857687	4.960169	5.038116
H	1.002782	6.055957	3.914273
H	4.696526	6.103285	10.150262
H	7.759078	17.029925	8.874259
H	8.020373	18.719654	8.363010
H	9.390603	17.589045	8.418689
H	5.833625	17.118133	5.042470

H	5.926468	18.613282	6.012137
H	5.491237	17.061846	6.781537
H	1.460874	15.218260	-0.869759
H	2.664888	16.067269	-1.871248
H	2.723203	16.204473	-0.096304
H	1.669165	3.505990	3.189126
H	1.690022	1.735214	3.002263
H	2.345433	2.775346	1.713934
H	5.691578	6.594534	1.518530
H	7.179550	5.998303	0.745137
H	7.092784	6.172556	2.513515
H	9.359579	10.218259	7.897132
H	7.590036	10.069507	8.072638
H	8.351949	9.716833	6.512446
H	4.288310	2.774353	0.287973
H	5.143403	4.011901	-0.659046
H	3.785844	4.483628	0.396694
H	4.842701	0.886477	1.948719
H	4.117496	0.070975	3.351333
H	5.745863	0.789829	3.483647
H	8.147394	2.523579	6.590139
H	6.428843	2.950205	6.835829
H	7.692016	4.148591	7.183562
H	9.681171	7.597226	6.188413
H	9.943764	6.471010	7.547713
H	10.760841	8.062090	7.525203
H	8.010838	9.837710	11.898536
H	6.847626	11.079185	11.376235
H	7.986542	10.381419	10.202662
H	8.044176	4.551390	9.223912
H	9.157480	4.266278	10.586368
H	9.466326	5.593033	9.431700
H	4.323960	7.728348	12.112772
H	4.606550	9.417002	12.609912
H	5.763957	8.152267	13.078944
H	9.532067	7.804223	11.626408
H	9.484019	6.431481	12.765907
H	8.328242	7.769827	12.942338
H	2.893678	5.794778	7.681845
H	2.874652	7.146030	8.841158
H	2.373705	5.516228	9.366881
H	5.317065	9.571458	8.370595
H	4.555590	10.540878	9.648074
H	3.958227	8.908816	9.282930
H	4.087209	3.714551	9.792252
H	5.794807	3.984054	9.337996

H	4.557474	3.845074	8.072565
H	5.939588	5.782827	12.844008
H	7.192138	4.516515	12.843971
H	5.903286	4.497586	11.614801

References

(1) Pal, P. K.; Chowdhury, S.; Drew, M. G. B.; Datta, D. The Electrooxidation of the Tetraphenylborate Ion Revisited. *New J. Chem.* **2002**, *26*, 367–371.
